# Natural Antioxidants Reduce Oxidative Stress and the Toxic Effects of RNA-CUG_(exp)_ in an Inducible Glial Myotonic Dystrophy Type 1 Cell Model

**DOI:** 10.3390/antiox14030260

**Published:** 2025-02-25

**Authors:** Fernando Morales, Dayana Vargas, Melissa Palma-Jiménez, Esteban J. Rodríguez, Gabriela Azofeifa, Oscar Hernández-Hernández

**Affiliations:** 1 Instituto de Investigaciones en Salud (INISA), Universidad de Costa Rica, San José 2060, Costa Rica; dayanna.vargassanabria@ucr.ac.cr (D.V.); melissamaria.palma@ucr.ac.cr (M.P.-J.); esteban.rodriguez_r@ucr.ac.cr (E.J.R.); 2 Departamento de Bioquímica, Escuela de Medicina, Universidad de Costa Rica, San José 2060, Costa Rica; gabriela.azofeifacordero@ucr.ac.cr; 3 Laboratorio de Medicina Genómica, Departamento de Genética, Instituto Nacional de Rehabilitación Luis Guillermo Ibarra Ibarra, INR-LGII, Mexico City 14389, Mexico; heroscar@gmail.com

**Keywords:** myotonic dystrophy, RNA toxic gain-of-function, inducible model, glial cell model, antioxidants, treatment

## Abstract

The toxic gain-of-function of RNA-CUG_(exp)_ in DM1 has been largely studied in skeletal muscle, with little focus on its effects on the central nervous system (CNS). This study aimed to study if oxidative stress is present in DM1, its relationship with the toxic RNA gain-of-function and if natural antioxidants can revert some of the RNA-CUG_(exp)_ toxic effects. Using an inducible glial DM1 model (MIO-M1 cells), we compared OS in expanded vs. unexpanded cells and investigated whether antioxidants can mitigate OS and RNA-CUG_(exp)_ toxicity. OS was measured via superoxide anion and lipid peroxidation assays. RNA foci were identified using FISH, and the mis-splicing of selected exons was analyzed using semi-quantitative RT-PCR. Cells were treated with natural antioxidants, and the effects on OS, foci formation, and mis-splicing were compared between treated and untreated cells. The results showed significantly higher superoxide anion and lipid peroxidation levels in untreated DM1 cells, which decreased after antioxidant treatment (ANOVA, *p* < 0.001). Foci were present in 51% of the untreated cells but were reduced in a dose-dependent manner following treatment (ANOVA, *p* < 0.001). Antioxidants also improved the splicing of selected exons (ANOVA, *p* < 0.001), suggesting OS plays a role in DM1, and antioxidants may offer therapeutic potential.

## 1. Introduction

Myotonic dystrophy type 1 (DM1) is a highly variable inherited disorder primarily characterized by myotonia, cataracts, muscle weakness and wasting, cardiac conduction defects, and central nervous system (CNS) involvement [1]. The mutation responsible for DM1 resulted in an unstable expansion of CTG repeats in the 3′ UTR of the *DMPK* gene [2,3,4], with symptomatic patients typically carrying more than 50 repeats. The DM1 pathomechanism is driven by a toxic gain-of-function of expanded RNA-CUG repeats (RNA-CUG_(exp)_) [5,6]. These expanded RNAs accumulate in the nucleus, forming foci (ribonuclear inclusions) that lead to (1) sequestration of the MBNL (muscleblind-like) family of proteins, which colocalize with RNA foci; and (2) an increase in CELF protein levels (which do not colocalize with the foci) [7,8]. Both protein families play crucial roles in regulating alternative splicing, particularly during the fetal-to-adult splicing transition [6,9,10]. The functional downregulation of MBNL and the upregulation of CELF (proteins with opposing actions [11,12,13]) result in widespread splicing abnormalities across many genes [14,15]. In adult DM1 patients, embryonic isoforms are expressed instead of the adult versions, highlighting the central role of this splicing dysregulation in DM1’s clinical presentation [16,17]. However, only a few of the numerous mis-splicing events identified in DM1 have been directly linked to specific symptoms. For example, myotonia is associated with the mis-splicing of exon 7a of the *CLCN1* gene [18,19], muscle weakness with the mis-splicing of exon 11 of *BIN1* and exon 29 of *CACNA1S* [20,21], cardiac arrhythmias with exon 5 of *cTNT* and exon 6 of *SCN5A* [22,23], and insulin resistance with exon 11 of *INSR* [24]. Given the complexity of the DM1 clinical picture and the involvement of various tissues and systems, additional cellular processes are likely to contribute to the disease’s pathology and variability [25]. One such process is oxidative stress (OS).

Reactive oxygen species (ROS) are essential in maintaining cellular homeostasis, differentiation, migration, and proliferation [26,27]. However, excessive ROS can lead to OS, damaging nucleic acids, proteins, and lipids and triggering inflammation [28,29,30,31,32,33], cell damage, and dysfunction [34,35,36]. OS is implicated in neurodegeneration and the development of neurodegenerative diseases such as Alzheimer’s disease (AD), Parkinson’s disease (PD), amyotrophic lateral sclerosis (ALS) [37,38,39], and repeat expansion disorders like spinocerebellar ataxia 7 (SCA7) and Friedreich’s ataxia (FA) [40,41,42,43,44]. In DM1, several studies have shown that OS biomarkers are elevated in patients compared to controls, and DM1 cell models (both neuronal and muscle) exhibit increased susceptibility to OS [45,46,47,48,49,50,51,52].

Although DM1 is classified as a muscular dystrophy due to its primarily muscle-related symptoms, recent findings suggest that CNS-related symptoms are equally, if not more, disabling [53,54,55]. As a result, DM1 is increasingly recognized as a neurodegenerative disease. Although brain cell death is not a hallmark of DM1, there is evidence of neurodegeneration [56,57,58]. Therefore, OS likely plays a key role in DM1 pathogenesis, similar to other repeat expansion diseases involving the CNS. Behavioral changes, reduced initiative, apathy, excessive daytime sleepiness, and fatigue are common in DM1 patients, and while there is no direct evidence linking these symptoms to OS in DM1, several reports suggest a possible association [59,60,61]. This implies that OS may contribute to some of the symptoms and disease progression in DM1 [49,50]. Thus, the aim of the study was to analyze OS in DM1, its relationship with the toxic RNA gain-of-function, and determine if natural antioxidants were able to revert some of the RNA-CUG_(exp)_ toxic effects. To analyze this, we used an in vitro inducible glial DM1 cell model using the MIO-M1 cell line [55] and explored whether certain hallmarks of toxic RNA-CUG_(exp)_ were related to OS and whether these hallmarks could be mitigated by treating cells with natural antioxidants.

## 2. Materials and Methods

### 2.1. Cell Culture, DOX Induction, and Antioxidant Treatment

A previously established inducible glial DM1 cell model (MIO-M1 line carrying 648 CTG repeats [MIO-M1 CTG_(648)_] [55]) was used in this project. Cells were cultured in DMEM supplemented with 10% FBS, 375 μg/mL G-418, 100 U/mL penicillin, 100 μg/mL streptomycin, and 0.17 μg/mL puromycin, with media changes every two days. Expression of the construct containing the CTG repeat expansion was induced by adding 1.0 μg/mL of doxycycline (DOX) to the culture media (uninduced cells were used as controls). The expression of the construct was confirmed as previously described [55]. Uninduced cells do not express the construct and are, therefore, considered as lacking the CTG repeat expansion. When required, to reduce cell detachment, a coating of 0.1 mg/mL Poly-D-Lysine (Merck, Rahway, NJ, USA) and 10 μg/mL laminin (Sigma Aldrich, St. Louis, MO, USA) was applied prior to seeding.

When cultures reached 80–90% confluence, antioxidants were added to the culture media. A day after adding antioxidants to the cultures, culture media was changed, and antioxidants were added again, plus DOX in order to induce the expression of the construct containing the CTG repeat expansion. Media (with antioxidants and DOX) was refreshed every two days for a total of four days. Control cells were kept untreated and uninduced. As antioxidants, we used polyphenol extracts from *Rubus adenotrichos* (blackberry) and *Bactris guineensis* (güiscoyol) as natural antioxidants, whose antioxidant properties had been previously tested, along with N-acetyl-L-cysteine (NAC) [62,63,64], a well-known FDA-approved antioxidant. The methodology for purifying the polyphenol extracts, as well as the recollection sites of the fruits, were reported previously [62,64]. In this study, we used the same extracts that had already been prepared for the mentioned studies. Three different concentrations of each antioxidant were tested: 7.5, 15, and 30 μg/mL for blackberry; 5, 10, and 15 μg/mL for güiscoyol; and 1, 3, and 5 mM for NAC. Cultures were grown in triplicate (or in duplicate, as indicated in the text) and incubated at 37 °C with 5% CO_2_. In each experiment, the different antioxidant treatments were compared to untreated DOX-induced MIO-M1 CTG_(648)_ expanded cells, while uninduced MIO-M1 CTG_(648)_ cells served as controls. As untreated controls, we used: (1) Positive control–MIO-M1 CTG_(648)_ cells induced with DOX without antioxidants; (2) Negative control–MIO-M1 CTG_(648)_ cells without DOX (uninduced) and without antioxidants. Treated conditions mimicked the positive control, but with antioxidants added to the culture media.

### 2.2. Measurement of Superoxide Anion and Lipid Peroxidation (LP)

To measure superoxide anion (the predominant intracellular ROS) in the MIO-M1 cell line, 3000 cells were seeded per well in a 96-well black plate, in duplicates, and cultured as previously described. Superoxide levels were assessed using the MitoSOX Red probe (Invitrogen^TM^, Waltham, MA, USA), a fluorogenic dye that emits a strong signal upon reacting with superoxide. Hoechst 33342 (Invitrogen^TM^, USA) was also used to stain the nuclei, aiding in image analysis. On the final day of antioxidant treatment, cells were washed with PBS, and the probes were added to each well (both treatments and controls) for 20 min at 37 °C, following the manufacturer’s instructions. The probes were prepared in culture media (MitoSOX 5 μM and Hoechst 33342 4 μg/mL). After incubation, cells were washed, and colorless media was added to each well for image analysis. Images were acquired by taking four photos per well using a cell imaging station (Cytation 3, BioTek Instruments, Winooski, VT, USA), and the images were processed using CellProfiler v4.2.6 software to quantify fluorescence in individual cells (on average, around 28 cells per photo). Cell fluorescence averages were obtained for each photo and compared across conditions. The experiments were performed twice at separate times.

Excess ROS can lead to OS, which is often indicated by LP. To confirm the presence of OS in the DM1 MIO-M1 inducible cell model and evaluate antioxidant effects on reducing OS, we quantified LP using the Nonyl Acridine Orange (NAO) probe (Sigma, Burbank, CA, USA). NAO detects LP in mitochondria by emitting a fluorogenic signal upon interacting with oxidized lipids. For this quantification, 15,000 cells were seeded per well in a 96-well black plate, in duplicates, and cultured as described above. On the final day of antioxidant treatment, cells were washed with PBS, and NAO (0.5 μg/mL) was added to each well (including both treatments and controls) for 30 min at 37 °C. After incubation, cells were washed, colorless media was added, and total fluorescence was measured at 491 nm/520 nm (Cytation 3, BioTek Instruments, Winooski, VT, USA). The experiment was performed twice, resulting in quadruplicate analyses for each condition.

### 2.3. Foci Detection by Fluorescent In Situ Hybridization (FISH) and MBNL1/2 Colocalization

To assess the presence of sense RNA foci, a hallmark of DM1, cells (both treated and control groups) were grown on slides in duplicate, and the experiment was repeated twice at different time points. Culture conditions were as previously described. Cells were washed three times with 1X PBS and fixed with 4% PFA at room temperature for 15 min. Afterward, cells were washed seven times with 1X PBS and treated with pre-cooled 2% acetone in 1X PBS for 5 min at 4 °C, followed by rehydration in 30% formamide/2X SSC buffer for 10 min at room temperature. Cells were then washed with 1X PBS for 5 min and incubated in prehybridization buffer for 30 min at 37 °C in a humid chamber while preparing the probe. Next, the cells were washed three times with 30% formamide/2X SSC, incubating at 37 °C between washes. Cells were then incubated in hybridization buffer containing approximately 50 ng of the (CAG)_8_ Cy3 probe for 2 h at 50 °C in a humid chamber. After hybridization, the slides were washed three times with pre-heated 30% formamide/2X SSC at 50 °C, followed by three washes with 1X SSC at room temperature. Direct immunofluorescence was then performed to assess colocalization with MBNL1/2. Cells were washed with 1X PBS, blocked with PBS++ (1.44 mL of 1X PBS, 15 µL of Triton X-100, 45 µL of Standardized Goat Serum) for 30 min at room temperature in a humid chamber, and incubated overnight at 4 °C with unconjugated anti-MBNL1 or anti-MBNL2 primary antibodies (1:200 in PBS++) (Invitrogen^TM^, USA). The samples were washed three times for 5 min in 1X PBS in a humid chamber and in the dark, then incubated at room temperature for 1 h with Alexa Fluor 488-conjugated anti-rabbit secondary antibody (1:500 in PBS++) (Invitrogen^TM^, USA). Cells were washed three times for 5 min with DEPC water, air-dried at 25 °C for 1 h, and a mounting medium containing DAPI was added before sealing the coverslips with clear commercial enamel. Slides were incubated at 4 °C for 24 h before being observed using an Olympus BX43 fluorescence microscope (Olympus Life Science, Waltham, MA, USA). To determine the percentage of cells with foci in each condition, at least 10 different optical fields, each containing 15–20 cells, were photographed using Life Science Imaging v1.6 software and edited using ImageJ package. The percentage of cells with foci in each optical field was calculated using the following formula: (# of cells with foci (positive cells)/total # of cells) * 100. The overall percentage of cells with foci per condition was obtained by averaging the data from each optical field. For colocalization analysis, three different optical fields were analyzed, and images were processed and merged using the ImageJ v1.54 package.

### 2.4. Mis-Splicing Analysis of Five Specific Exons by Semi-Quantitative RT-PCR

Cells were cultured in triplicate, and due to the observed dose-dependent gradient of antioxidant effects in our model, we used only the highest and lowest antioxidant concentrations. Total RNA was purified using TRIzol (Life Technology, Invitrogen^TM^, USA) and the PureLink RNA Mini Kit (Ambion, Austin, TX, USA), following the manufacturer’s instructions. RNA was quantified using a NanoDrop spectrophotometer (Thermo Scientific, Waltham, MA, USA), its integrity was assessed on a 1% agarose gel, and it was stored at −80 °C. Approximately 500 ng of RNA per culture condition was treated with DNase and reverse transcribed using the SuperScript IV VILO Master Mix (Invitrogen^TM^, USA) according to the manufacturer’s protocol. The resulting cDNA was treated with RNase A and stored at −20 °C. Alternative splicing of candidate exons was evaluated by PCR (two PCRs per culture, totaling six PCRs per condition), using the RNA polymerase II subunit A (*POLR2A*) gene as an internal control. Standard PCR conditions were used to amplify the *POLR2A* gene (Supplementary Materials). PCR products were resolved on agarose gels and quantified using Image Lab software 6.1 (Bio-Rad, Hercules, CA, USA) (all gel images are included in Supplementary Materials). To ensure consistency in the amount of cDNA used for PCR, the *POLR2A* PCR was repeated until the same amount of cDNA was obtained for each culture condition (Figure S1). PCR conditions and primers for each splicing event analyzed in this study were used as previously described (Supplementary Materials). The splicing events analyzed included: exon 7 of *MBNL1* and *MBNL2* genes [55], exon 8 of *APP* gene [65], exon 27 of *ITGA6* gene [66] and exon 30 of *SORBS1* gene [66]. PCR products were resolved on 2% agarose gels stained with ethidium bromide. Quantification of the PCR products was carried out using Image Lab software (Bio-Rad, USA). The percentage of exon inclusion (PSI-ψ) was calculated as: [exon inclusion band/(exon inclusion band + exon exclusion band)] × 100.

### 2.5. Analysis

Statistical analyses were performed using SPSS 20 software. Data are presented as mean ± standard error of the mean (±SEM). For comparing between conditions, we performed one-way ANOVA (Tukey’s post hoc test was used for multiple comparisons). The Mann–Whitney U (MWU) rank sum test was used to compare the median of two variables. Results were considered significant when *p* ≤ 0.05.

### 2.6. Artificial Intelligence

We took advantage of GenAI to improve English writing and translation of some parts of the manuscript to make the reading more fluent.

## 3. Results

### 3.1. Natural Antioxidants Counteract the OS Observed in an Inducible DM1 Glial Cell Model

**Figure 1:**
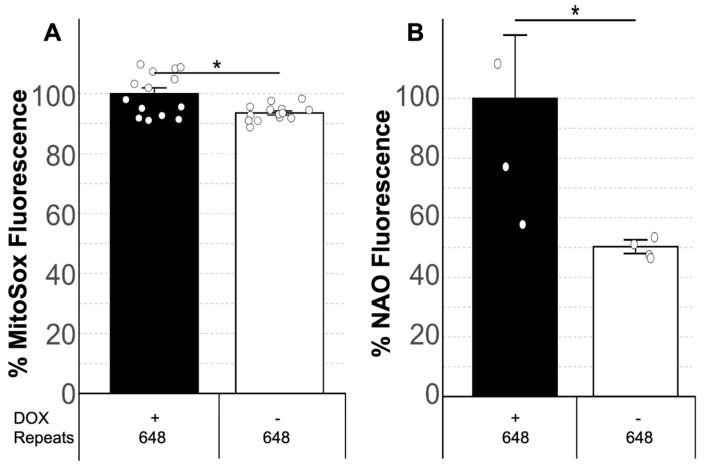
OS in a DM1 glial cell model. Plot (**A**) shows the results of quantifying the levels of superoxide anion (% MitoSox Fluorescence) in MIO-M1 CTG_(648)_ in the presence (+) or absence (−) of DOX. The plot shows that levels of superoxide anion are significantly increased in induced cells compared to uninduced cells. Plot (**B**) shows the results of quantifying the levels of LP (% NAO Fluorescence) in MIO-M1 CTG_(648)_ in the presence (+) or absence (−) of DOX. The plot shows that levels of LP are significantly increased in induced cells compared to uninduced cells. * = MWU, *p* < 0.05.

**Figure 2:**
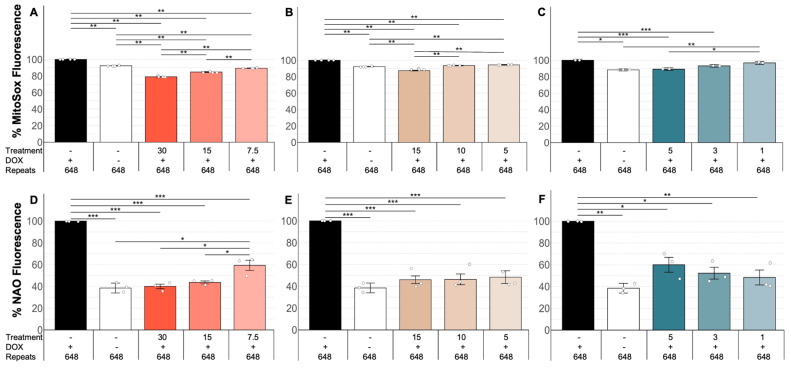
Natural antioxidants reduce OS in a DM1 glial cell model. Plots (**A**–**C**) show the results of quantifying the superoxide anion (% MitoSox Fluorescence) in MIO-M1 CTG_(648)_ in the presence (+) or absence (−) of DOX and different concentrations of antioxidants (described in the text). As can be seen, levels of superoxide anion are significantly increased due to the CTG repeat expansion (two first columns), which are significantly decreased after treating the cells with polyphenol extracts from *Rubus adenotrichos* (**A**), from polyphenol extracts from *Bactris guineensis* (**B**), and NAC (**C**). A dose-dependent effect of the antioxidant is seen for all treatment conditions. Plots (**D**–**F**) show the results of quantifying lipid peroxidation in the glial cell model under study. As can be seen, levels of LP are significantly increased due to the CTG repeat expansion (two first columns), which are significantly decreased after treating the cells with polyphenol extracts from *Rubus adenotrichos* (**D**), from polyphenol extracts from *Bactris guineensis* (**E**), and NAC (**F**). A dose-dependent effect of the antioxidant is seen for antioxidants except from NAC (**F**). To allow a proper comparison, data were given as a percentage. ANOVA (Tukey), * = *p* < 0.05, ** = *p* < 0.001, *** = *p* < 0.0001.

In order to analyze OS in DM1, we quantified ROS levels—particularly superoxide anions, which are the primary oxygen radicals in cells and trigger the formation of many other ROS [67]—and LP levels, as both have been described as reliable OS biomarkers [28,29,30,31]. To investigate this, we quantified superoxide and LP levels in induced versus uninduced MIO-M1 CTG_(648)_ cells. Interestingly, we found that superoxide levels were significantly higher in induced MIO-M1 CTG_(648)_ cells compared to their uninduced counterparts (Figure 1A, MWU, *p* = 0.01). We also observed a significant increase in LP levels in the induced cells relative to the uninduced ones (Figure 1B, MWU, *p* = 0.02), suggesting that OS is linked to the CTG repeat expansion. Next, we explored whether natural antioxidants could reduce superoxide and LP levels and, consequently OS. To test this, cells were treated with three different concentrations of polyphenol extracts from two Costa Rican fruits—*Rubus adenotrichos* (a type of tropical highland blackberry) and *Bactris guineensis* (a tropical endemic palm commonly known as güiscoyol)—as well as NAC, a well-known antioxidant. Notably, we observed that all three antioxidants significantly reduced both superoxide levels (Figure 2A–C, ANOVA, *p* < 0.05) and LP levels (Figure 2D–F, ANOVA, *p* < 0.05) in induced MIO-M1 CTG_(648)_ cells compared to untreated induced cells, in a dose-dependent manner, except for LP levels with NAC.

### 3.2. Natural Antioxidants Reduce the Percentage of Cells with Foci in an Inducible DM1 Glia Cell Model

**Figure 3:**
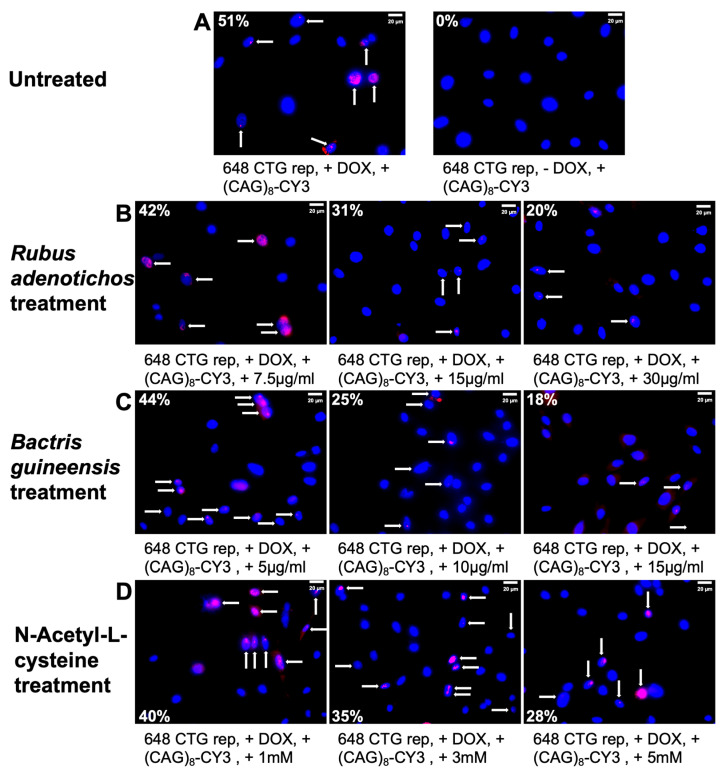
Foci formation and antioxidant treatment. The panel shows the percentage of cells with foci in the inducible glial DM1 cell model under study and the effect of the antioxidants on foci accumulation. MIO-M1 CTG_(648)_-induced cells without antioxidant treatment ((**A**)—top-left panel) show 51% of cells with foci, which is decreased after treating these cells with polyphenol extracts from *Rubus adenotrichos* ((**B**)—going down by up to 20%), polyphenol extracts from *Bactris guineensis* ((**C**)—going down by up to 18%), and NAC ((**D**)—going down by up to 28%). MIO-M1 CTG_(648)_ uninduced cells as a negative control of antioxidant treatment (top-right) show 0% of cells with foci. A dose-dependent effect of the antioxidant is seen in each treatment condition ((**B**–**D**)—a decrease in the % of cells with foci from left to right). Arrows indicate the presence of cells with foci (nuclei stained with DAPI).

**Figure 4:**
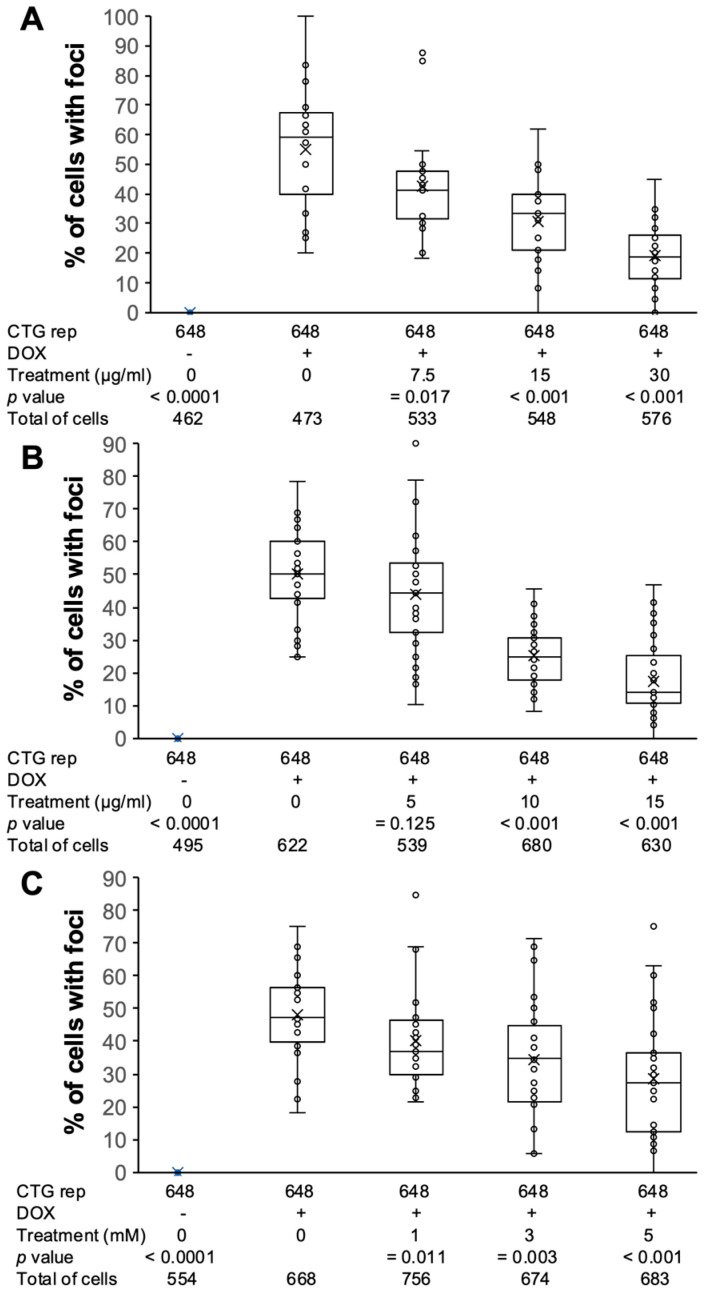
Relationship between foci formation and antioxidant treatment. The three plots show a negative relationship between the percentage of cells with foci and the concentration of the antioxidant used, (**A**)—polyphenol extracts from *Rubus adenotrichos*; (**B**)—polyphenol extracts from *Bactris guineensis*; and (**C**)—NAC, indicating a dose-dependent effect of the antioxidant (decrease in the % of cells with foci as the concentration of the antioxidant increases). Analyses (ANOVA-Tukey) indicate a significant difference in the % of cells with foci in the treated conditions compared to untreated cells, especially with the two highest antioxidant concentrations.

Taking advantage of the fact that the MIO-M1 CTG_(648)_ model recapitulates some of the key features of DM1 [55], such as RNA foci accumulation, we sought to determine whether natural antioxidants could reduce the percentage of DM1 cells with RNA foci. We conducted fluorescence in situ hybridization (FISH) and quantified the percentage of cells with foci. As expected, induced MIO-M1 CTG_(648)_ cultures showed 51% of cells with foci, while uninduced cultures showed 0% (Figure 3A). After confirming foci accumulation, we treated MIO-M1 CTG_(648)_ cells with natural antioxidants at three different concentrations and measured the percentage of cells with foci following treatment. Remarkably, after treatment with (1) polyphenol extracts from *Rubus adenotrichos*, we observed a reduction in foci accumulation from 51% to 20% (Figure 3B and Figure 4A); (2) polyphenol extracts from *Bactris guineensis*, foci accumulation decreased from 51% to 18% (Figure 3C and Figure 4B); and (3) NAC, foci accumulation dropped from 51% to 28% (Figure 3D and Figure 4C). In all three treatments, the reduction in foci was dose-dependent, indicating a negative correlation between foci accumulation and antioxidant concentration—higher antioxidant concentrations led to a greater, significant reduction in cells with foci (Figure 4, ANOVA, *p* < 0.05). However, it is possible that antioxidants may inhibit the expression of toxic RNA rather than disrupting already-formed foci. To test this, we repeated the FISH experiment, but this time, we first induced the expression of the construct with DOX, and then, we treated the cells with antioxidants (we used only the highest concentration). We analyzed about 10 different optical fields and calculated the percentage of cells with foci per condition. Our results show that the percentage of cells with foci significantly decreased to around 15% (Figure S2, ANOVA, *p* < 0.0001), consistent with previous results (Figure 3 and Figure 4), and confirming the protective effects of the tested antioxidants (Figure S2).

### 3.3. Natural Antioxidants Rescue Some Mis-Splicing Events Observed in an Inducible DM1 Glial Cell Model

**Figure 5:**
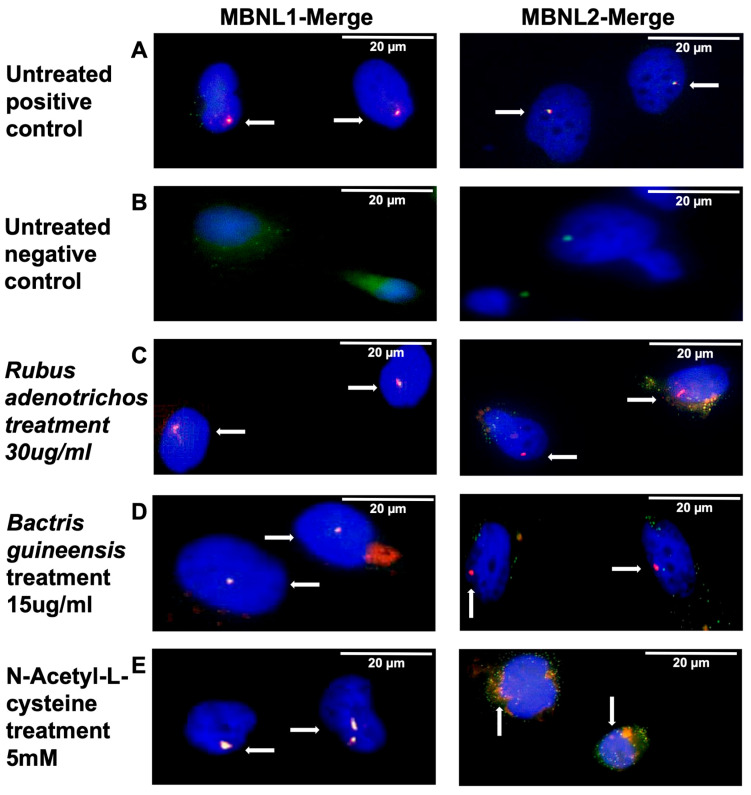
MBNL1/2 colocalization and antioxidant treatment. These panels correspond to a magnification of panels shown in Figure S3. The panels show MBNL1 (left panels) and MBNL2 (right panels) foci colocalization in different culture conditions. (**A**)—MIO-M1 CTG_(648)_-induced cells without antioxidant treatment. Colocalization is observed for both MBNL1 and MBNL2. (**B**)—MIO-M1 CTG_(648)_-uninduced cells without antioxidant treatment. Colocalization is not observed for both MBNL1 and MBNL2. (**C**)—MIO-M1 CTG_(648)_-induced cells treated with the highest concentration of polyphenol extracts from *Rubus adenotrichos*. Clearer colocalization is observed for MBNL1 but not for MBNL2. (**D**)—MIO-M1 CTG_(648)_-induced cells treated with the highest concentration of polyphenol extracts from *Bactris guineensis*. Clearer colocalization is observed for MBNL1 but not for MBNL2. (**E**)—MIO-M1 CTG_(648)_-induced cells treated with the highest concentration of NAC. Clearer colocalization is observed for MBNL1 but not for MBNL2. Arrows show some of the signals corresponding to colocalization or the absence of colocalization.

Another hallmark of DM1 observed in MIO-M1 DM1 cells is the mis-splicing of specific exons, such as *MBNL1* exon 7 and *MBNL2* exon 7 [55]. It is well-known that foci formation and colocalization with MBNL1/2 are key drivers of the splicing dysregulation seen in DM1 [68]. Therefore, reducing foci or disrupting this colocalization could be important for rescuing mis-splicing events. Before investigating mis-splicing rescue, we explored whether antioxidants could disrupt MBNL1/2 colocalization, as they did with foci accumulation. To do this, we performed FISH followed by direct immunofluorescence using specific anti-MBNL1 and anti-MBNL2 antibodies. In untreated induced MIO-M1 CTG_(648)_ cells, we observed clear MBNL1 and MBNL2 foci-colocalization (Figure 5A, Figure S3, Supplementary Materials), which was absent in untreated uninduced cells (Figure 5B, Figure S3, Supplementary Materials). Following antioxidant treatment, we found that while the highest concentrations of antioxidants did not seem to disrupt MBNL1 foci-colocalization (Figure 5C–E, Figure S3, Supplementary Materials), they did seem to disrupt MBNL2 foci-colocalization, as the MBNL2 signal seemed more dispersed (Figure 5C–E, Figure S3, Supplementary Materials). Due to space constraints, Figure 5 shows only the merged images with the highest antioxidant concentrations, but all images (DAPI, (CAG)_8_ Cy3 probe, anti-MBNL1/2, Merge) for all conditions can be found in the Supplementary Materials.

**Figure 6:**
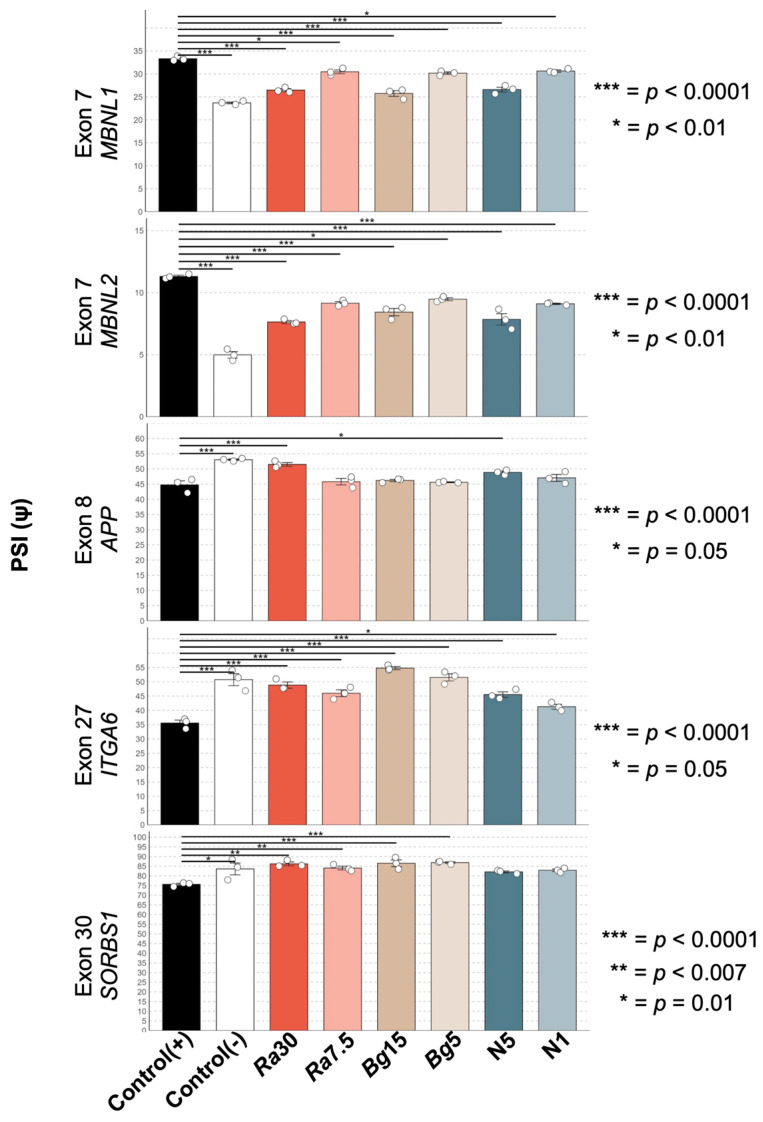
DM1 splicing rescued by antioxidant treatment. The figure shows the PSI of five different alternative exons (labeled on the left-hand side of each plot) analyzed by different culture conditions (columns labeled at the bottom of the figure). When compared to the uninduced MIO-M1 CTG_(648)_ cells (second column–control (−)), we observed in the induced MIO-M1 CTG_(648)_ cells (first column–control (+)) a significantly increased exon inclusion for exons in *MBNL1/2* genes; while for the other three exons analyzed, we observed, in the positive control, a significantly increased exon exclusion. After treating the cells with polyphenol extracts from *Rubus adenotrichos* (columns *Ra*30 and *Ra*7.5), polyphenol extracts from *Bactris guineensis* (columns *Bg*15 and *Bg*5), and NAC (columns N5 and N1) we observed a significant rescue in the PSI for most of the exons in most of the conditions and observed a dose-dependent effect (only the highest and lowest concentration of the antioxidant were tested). Statistical analysis was carried out using ANOVA (Tukey); *p*-values are on the right-hand side of each plot.

Since the percentage of cells with foci was reduced, and MBNL2 foci-colocalization seemed disrupted in induced MIO-M1 CTG_(648)_ cells treated with antioxidants, we hypothesized that this might lead to a rescue of some mis-splicing events. To test this, we analyzed alternative splicing patterns before and after antioxidant treatment in MIO-M1 CTG_(648)_ cells. The target exons included exon 7 of *MBNL2*, exon 8 of *APP*, exon 7 of *MBNL1*, exon 27 of *ITGA6*, and exon 30 of *SORBS1*. For all five exons, the splicing patterns matched those previously described in the DM1 context: reduced inclusion of exons in *SORBS1*, *ITGA6*, and *APP* and increased inclusion of exons in *MBNL1* and *MBNL2*. These differences in the percent spliced-in (PSI) values between induced and uninduced MIO-M1 CTG_(648)_ cells were statistically significant (ANOVA, *p* < 0.05, Figure 6, columns one (positive control [+]) and two (negative control [−])). Remarkably, antioxidant treatment significantly rescued the splicing of all five exons in a dose-dependent manner (ANOVA, *p* < 0.05, Figure 6). However, not all antioxidants were equally effective in rescuing splicing events: *APP* exon 8 was rescued only by the highest concentrations of *Rubus adenotrichos* extract and NAC (Figure 6). In some cases, the rescue of mis-splicing approached PSI levels similar to those of uninduced MIO-M1 CTG_(648)_ cells, particularly for exon 30 of *SORBS1* and exon 7 of *MBNL1* (Figure 6). Collectively, our results suggest that OS occurs in the MIO-M1 DM1 model, and more importantly, some of the toxic effects triggered by RNA-CUG_(exp)_ can be reversed by antioxidant treatment.

## 4. Discussion

OS appears to be a common feature in many human conditions, both hereditary—such as metabolic and neurodegenerative diseases—and non-hereditary, including most cancers. Its multiple effects, which extend beyond the imbalance of specific molecules, include cell damage, inflammation, and biomolecule damage, all of which are key contributors to disease pathology [37,38,39]. OS has been reported in diseases such as Alzheimer’s disease (AD), Parkinson’s disease (PD), amyotrophic lateral sclerosis (ALS), and several repeat expansion disorders [40,41,42,43,44], including DM1 [45,46,47,48,49,50,51,52]. In the case of DM1, the few studies available have focused on redox imbalances, cytotoxicity, and OS biomarkers in DM1 patients and cell models [46,47,49,50]. However, the relationship between OS and the DM1 mutation, particularly the toxic RNA-CUG_(exp)_ gain-of-function, remains unclear.

To explore the potential relationship between OS and the DM1 mutation, we cultured cells in which the only difference was the presence of the CTG repeat expansion (i.e., induced vs. uninduced MIO-M1 CTG_(648)_ cells). Our results showed that ROS levels (particularly superoxide) and LP were elevated in induced MIO-M1 CTG_(648)_ cells (Figure 1), consistent with previous findings in patients and other models [45,46,47,48,49,50,51,52]. Since both ROS and LP are biomarkers of OS, this suggests that OS is a prominent feature in DM1 and might be a consequence of the DM1 mutation. However, the extent of OS’s contribution to DM1 pathology and which specific symptoms it explains are still unclear, though it has been speculated that OS is related to disease progression [49]. Further studies analyzing OS and cell/animal models, or even DM1 patients carrying different expansion sizes, could contribute to understanding how OS is related to DM1 pathology, mainly due to it is possible that different CTG repeat expansion sizes may trigger varying levels of OS biomarkers, which could mean that the role of OS in DM1 varies among patients. This idea is not unreasonable, given that CTG repeat size plays a crucial role in DM1. For example, the estimated progenitor allele length (ePAL)–the size of the allele transmitted from an affected parent to their offspring–is a major modifier of disease severity, age of onset, somatic instability, and hypermethylation of regions flanking the CTG repeat [69,70,71,72]. This clearly demonstrates that many of the clinical and molecular features observed in DM1 are likely a consequence of the expansion size.

Therefore, reducing OS could be beneficial for DM1 patients. The most direct way to counteract OS is typically through the use of antioxidants. In DM1, only a few studies have examined the use of antioxidants (mostly in cell models) for treatment purposes. Previous research has shown that certain flavonoids [52] and Trolox [48] effectively reduce OS, apoptosis, cytotoxicity, and *cis*-effects of the CTG repeat. In this study, we used polyphenol extracts from two tropical fruits (consumed often in tropical and other populations), *Rubus adenotrichos* (blackberry) and *Bactris guineensis* (güiscoyol), as a potential antioxidant therapy administrated alongside standard. Increasingly, the scientific literature and epidemiological studies have highlighted the potential of bioactive compounds in certain foods and their positive health effects beyond their basic nutritional value [73]. In particular, polyphenols have been associated with a reduced risk of several chronic diseases, including cancer, cardiovascular disease, and neurodegenerative diseases such as Alzheimer’s and Parkinson’s [74]. The polyphenol extracts used in this study contained ellagitannins and anthocyanins from the blackberry extract and proanthocyanidins from the güiscoyol extract. Both extracts have demonstrated antioxidant properties by reducing OS in other models, primarily due to the presence of hydroxyl substituents that donate hydrogen atoms and scavenge unpaired electrons, and as natural products, they do not represent any health risk to humans [62,75].

Using the same polyphenol extracts as in previous studies, our results clearly show that the OS observed in the glial DM1 cell model–possibly caused by the CTG repeat expansion–is reduced following treatment with natural polyphenol extracts (Figure 2A–C and Figure 2D–F). This was further confirmed by the fact that the antioxidant effect was dose-dependent, a finding demonstrated for the first time in this context. The antioxidant effect was evident not only in reducing free radical species (particularly superoxide) but also in mitigating the damage these oxidative compounds cause, as indicated by the reduction in LP markers.

Having established that OS is present in this glial DM1 cell model and that OS is reduced following treatment with natural antioxidants, we then explored whether there was a relationship between OS and the toxic RNA-CUG_(exp)_ gain-of-function. To investigate this, we assessed the percentage of cells with foci and analyzed specific mis-splicing events in the model, similar to previous studies on *MBNL1/2* exon 7 [55]. As expected, we observed a significant increase in the percentage of cells with foci in induced MIO-M1 CTG_(648)_ cells compared to uninduced control cells (55% vs. 0%, Figure 3 and Figure 4). Interestingly, after treating the MIO-M1 DM1 cells with natural antioxidants, the percentage of cells with foci significantly decreased in a dose-dependent manner, confirming the protective effects of the antioxidants (Figure 3, Figure 4 and Figure S2) and suggesting a possible relationship between OS and the toxic RNA-CUG_(exp)_ gain-of-function.

It is well-established that MBNL1 and MBNL2 colocalize with RNA foci, leading to mis-splicing of specific exons due to the reduced bioavailability of these proteins [9,12,76]. Our findings suggest that antioxidants might disrupt MBNL2 colocalization, implying that more MBNL proteins would be available (Figure 5, Figure S3, Supplementary Materials). Since antioxidants also reduce the percentage of cells with RNA foci in this glial DM1 model, it stands to reason that some mis-splicing events may be rescued. Indeed, we report for the first time that natural compounds can rescue the mis-splicing of specific exons in this model (Figure 6). However, the effects of antioxidants are not uniform across all exons. For example, the percent spliced-in (PSI) values for exon 7 in the *MBNL1* gene and exon 30 in the *SORBS1* gene were rescued almost to the levels seen in uninduced cells when treated with the highest concentration of antioxidants. In contrast, the rescue of other exons was more moderate, though still significantly different from untreated cells (Figure 6). This suggests that the efficacy of antioxidant treatment may vary depending on which exons or compounds are involved. Furthermore, individual variation in mis-splicing, previously described in DM1, may also play a role [77].

This raises two important questions: Could antioxidants produce similar effects in other DM1 cell types? And how might OS and the toxic RNA-CUG_(exp)_ gain-of-function be connected? The first question is difficult to answer now, as there have been few studies in this area. Most have used a limited number of cell types. Promising results have been seen in PC12 cells [52] and in our study using the MIO-M1 cell line, but there are no studies yet using DM1 patient-derived cells from tissues such as the brain or muscle. It is likely that antioxidant effects will vary by tissue, which underscores the need for further studies to identify effective treatments across different DM1 tissues.

Regarding the second question, this is the first study to suggest a relationship between OS and the toxic RNA-CUG_(exp)_ gain-of-function. While ROS are essential for many cellular processes under normal conditions, elevated levels of ROS lead to OS, which can disrupt various signaling pathways and transcription factors through phosphorylation. These include pathways such as mitogen-activated protein kinases (MAPKs) and PI3K/Akt, as well as transcription factors like Nrf2, HIF-1α, AP-1, and NF-κB [78]. Notably, in DM1, dysfunction was observed in several key signaling pathways, many of which are regulated by protein phosphorylation. Major pathways disrupted in DM1 include (1) AKT/mTOR, (2) AMPK, (3) PKC, and (4) MEK/ERK, along with downstream effectors like CUG-BP1 [79]. Both MBNL1 and CUG-BP1 are RNA-binding proteins that regulate alternative splicing, and both are dysregulated in DM1, contributing to the mis-splicing observed in various tissues [80]. Given that CUG-BP1 malfunction results from pathway dysregulation and that OS alters these pathways, it is possible that OS is responsible for CUG-BP1 dysfunction. By reducing OS, CUG-BP1 function could potentially be restored, thereby rescuing splicing. For example, CUG-BP1 promotes exon 8 exclusion in the APP gene [81], which aligns with our findings, although there is limited information on *ITGA6* and *SORBS1* exons. A similar mechanism may apply to MBNL proteins, although no direct evidence currently links MBNL to signaling proteins. It remains possible that OS affects MBNL function or that reducing OS with natural antioxidants alters the affinity between expanded RNA(CUG) repeats and RNA-binding proteins, as previously speculated [52]. Importantly, our results do not suggest a mechanistic link between OS and RNA-CUG_(exp)_ toxicity (this was not the scope of the study). Instead, it shows for the first time evidence of a possible relationship between those two variables. Now, with the evidence provided in this study, it might be possible to carry out in vivo studies to analyze this further.

Natural antioxidants have been explored as potential therapeutic options for a variety of diseases, particularly through antioxidant-rich diets. Thanks to the potent antioxidant and anti-inflammatory properties of polyphenols, studies have shown that these compounds can help attenuate muscle atrophy, protect neurons, and even enhance memory and cognitive functions [82,83,84,85]. Some polyphenols may also cross the blood–brain barrier (BBB), although this ability varies based on their structure [74]. However, clinical trials involving polyphenols are limited, and questions remain about appropriate dosages, potential side effects, and their efficacy and bioavailability in humans [86]. Nonetheless, an antioxidant-rich diet may improve clinical phenotypes over the medium and long term, potentially delaying disease onset and enhancing the quality of life for DM1 patients.

In summary, we demonstrated an apparently direct relationship between OS and the DM1 mutation in MIO-M1 CTG_(648)_ cells and that OS can be reduced with antioxidant treatment. For the first time, we also established a clear connection between OS and the toxic RNA-CUG_(exp)_ gain-of-function, showing that antioxidants reduce the effects of RNA(CUG) toxicity. As attractive as these results appear, in vivo and animal models are still required to confirm them, highlight OS as an important contributor to DM1 pathology, and confirm the use of antioxidants in DM1, which based on our results, could have the potential to serve as coadjuvants in DM1 therapeutic approaches.

## Data Availability

All data are shown in the results.

## Supplementary Materials

The following supporting information can be downloaded at: https://www.mdpi.com/article/10.3390/antiox14030260/s1, Supplementary Material: with details of the RT-PCR, Supplementary figures and material. Figure S1. cDNA homogenization. The picture shows two EtBr agarose gels showing RT-PCR products for *POLR2A* gene. Each lane corresponds to a PCR product for each culture for each condition, indicated above the gel image. Conditions used are: positive control (C+)—untreated induced MIO-M1 CTG_(648)_ cells; negative control (C-)—untreated uninduced MIO-M1 CTG_(648)_ cells (which was processed twice); *Ra*—induced MIO-M1 CTG_(648)_ cells treated with polyphenol extracts from *Rubus adenotrichos* at two concentrations, 7.5 μg/mL (*Ra*7.5) and 30 μg/mL (*Ra*30); *Bg*: induced MIO-M1 CTG_(648)_ cells treated with polyphenol extracts from *Bactris guineensis* at two concentrations, 5 μg/mL (*Bg*7.5) and 15 μg/mL (*Bg*30); N—induced MIO-M1 CTG_(648)_ cells treated with N-acetyl cysteine at two concentrations, 1mM (N1) and 5 mM (N5). B = no DNA. M = molecular weight marker. Figure S2. Foci formation and antioxidant treatment after induction. Cells were induced by 2 days, and after that, antioxidants were added to the culture and treated for 3 days. Foci detection and counting was performed as described in the main text. A: MIO-M1 CTG_(648)_ uninduced cells (negative control). B: MIO-M1 CTG_(648)_ induced cells (positive control) C: MIO-M1 CTG_(648)_ induced cells treated with the highest concentration (30 μg/mL) of polyphenol extracts from *Rubus adenotrichos*. D: MIO-M1 CTG_(648)_ induced cells treated with the highest concentration (15 μg/mL) of polyphenol extracts from *Bactris guineensis*. E: MIO-M1 CTG_(648)_ induced cells treated with the highest concentration (5mM) of NAC. The percentage of cells with foci is indicated on each micrography (bottom right), with arrows pointing out the foci. F: Box plot showing the statistical analysis (ANOVA-Tukey), which indicates that the antioxidants significantly reduce the percentage of cells with foci in cultures where antioxidants were added after DOX induction, *p* < 0.001. *R.a* (30) = *Rubus adenotrichos* at 30 μg/mL; *B.g* (15) = *Bactris guineensis* at 15 μg/mL; NAC (5) = NAC at 5mM. Figure S3. MBNL1/2 colocalization and antioxidant treatment. The panels show MBNL1 (left panels) and MBNL2 (right panels) foci colocalization in different culture conditions. A—MIO-M1 CTG_(648)_ induced cells without antioxidant treatment. Colocalization is observed for both, MBNL1 and MBNL2. B—MIO-M1 CTG_(648)_ uninduced cells without antioxidant treatment. Colocalization is not observed for both, MBNL1 and MBNL2. C—MIO-M1 CTG_(648)_ induced cells treated with the highest concentration of polyphenol extracts from *Rubus adenotrichos*. Clearer colocalization is observed for MBNL1 but not for MBNL2. D—MIO-M1 CTG_(648)_ induced cells treated with the highest concentration of polyphenol extracts from *Bactris guineensis*. Clearer colocalization is observed for MBNL1 but not for MBNL2. E—MIO-M1 CTG_(648)_ induced cells treated with the highest concentration of N-acetyl cysteine. Clearer colocalization is observed for MBNL1 but not for MBNL2. Arrows show some of the signals corresponding to colocalization or the absence of colocalization.
