# Supplementary material for: Natural Antioxidants Reduce Oxidative Stress and the Toxic Effects of RNA-CUG(exp) in an Inducible Glial Myotonic Dystrophy Type 1 Cell Model
Source: Antioxidants (Basel). 2025 Feb 25;14(3):260. doi: 10.3390/antiox14030260 (PMC11939792; doi:10.3390/antiox14030260)

Supplementary Material

Natural Antioxidants Reduce Oxidative Stress and the Toxic Effects of RNA-CUG$_{(exp)}$ in an Inducible Glial Myotonic Dystrophy Type 1 Cell Model

Fernando Morales [1,*] , Dayana Vargas [1], Melissa Palma-Jiménez [1] , Esteban J. Rodríguez [1] ,

Gabriela Azofeifa [2] and Oscar Hernández-Hernández [3]

1 Instituto de Investigaciones en Salud (INISA), Universidad de Costa Rica, San José 2060, Costa Rica

2 Departamento de Bioquímica, Escuela de Medicina, Universidad de Costa Rica, San José 2060, Costa Rica

3 Laboratorio de Medicina Genómica, Departamento de Genética, Instituto Nacional de Rehabilitación Luis Guillermo Ibarra Ibarra, INR-LGII, Mexico City 14389, Mexico

* Correspondence: fernando.moralesmontero@ucr.ac.cr

RT-PCR for the genes analyzed

Once cDNA was obtained following the protocol described in the main text, PCR was carried out following standard conditions. By using flanking primers in the region of interest, PCRs were performed with 1μM of each primer, 1X PCR buffer, 0.5 U *Taq* DNA polymerase and 2μl of cDNA in a total volume of 10μl. Annealing temperature and extension time varied according to the primers used to carry out the amplification, but in general temperatures were from 54 ºC to 62 ºC, and extension times were from 30 to 45 seconds. Reactions were cycled as follows: initial denaturing at 95 ºC for 10min, 27-35 cycles of denaturing temperature at 95 ºC for 30 seconds, annealing temperature at 54-62 ºC for 30 seconds, extension temperature at 72 ºC for 30-45 seconds and a final extension step of 72 ºC for 10 minutes. Details for individual genes analyzed can be found in the below table. All PCRs were carried out in a Biometra thermal cycler using 200μl tubes. Each reaction was covered with ~20 μl of white mineral oil. The thermal cycler lid was preheated to 105 ºC. Any additional information required can be requested.

Table: List of oligonucleotides and PCR conditions used in this project

Gene	Alt. exon	Size	Name	Sequence 5´ - 3´	Primer conc.	Annealing	Extension time	No of cycles
POLR2A			**POLR2A-F**	AGAGAAGCTGGTGCTCCGTA	1.0 µM	62°C	30 sec	30
			**POLR2A-F**	AGCGCAGGAAGACATCATCA				
SORBS1	30	168	**SORBS1-F**	CTGGGGATCTCACTAGCTTGGAG	1.0 µM	56 °C	45 sec	33
			**SORBS1-R**	GCCGTGGTGTTCTCCTTCATAC				
ITGA6	27	130	**ITGA6-F**	GAGTGACTGTGTTTCCCTCAAAGAC	1.0 µM	54 °C	45 sec	27
			**ITGA6-R**	CAGCCACGCCAAAAATAAAGG				
MBNL1	7	54	**MBNL1-F**	GCTGCCCAATACCAGGTCAAC	1.0 µM	62°C	30 sec	27
			**MBNL1-R**	TGGTGGGAGAAATGCTGTATGC				
MBNL2	7	54	**MBNL2-F**	ACAAGTGACAACACCGTAACCG	1.0 µM	62°C	30 sec	35
			**MBNL2-R**	TTTGGTAAAGGATGAAGAGCACC				
APP	8	57	**APP-F**	CCACAGAGAGAACCACCAGCATTGC	1.0 µM	57°C	30 sec	28
			**APP-R**	GATACTTGTCAACGGCATCAGGGG				

Supplementary Figures.

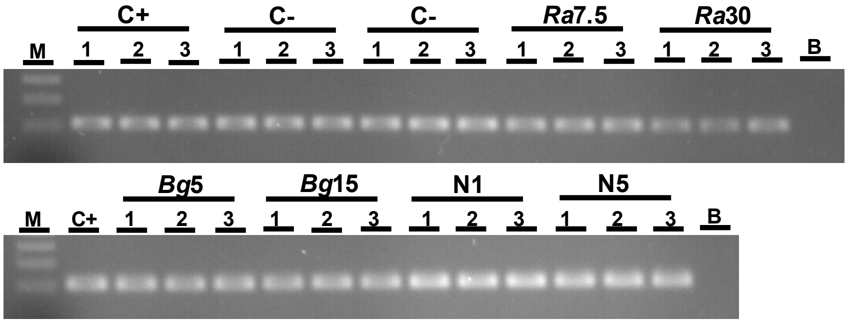

Figure S1. cDNA homogenization. The picture shows two EtBr agarose gels showing RT-PCR products for *POLR2A* gene. Each lane correspond to a PCR product for each culture for each condition, indicated above the gel image. Conditions used are: positive control (C+) – untreated induced MIO-M1 CTG$_{(648)}$ cells; negative control (C-) – untreated uninduced MIO-M1 CTG$_{(648)}$ cells (which was processed twice); *Ra* – induced MIO-M1 CTG$_{(648)}$ cells treated with polyphenol extracts from *Rubus adenotrichos* at two concentrations, 7.5µg/ml (*Ra*7.5) and 30 µg/ml (*Ra*30); *Bg*: induced MIO-M1 CTG$_{(648)}$ cells treated with polyphenol extracts from *Bactris guineensis* at two concentrations, 5µg/ml (*Bg*7.5) and 15 µg/ml (*Bg*30); N – induced MIO-M1 CTG$_{(648)}$ cells treated with N-acetyl cysteine at two concentrations, 1mM (N1) and 5 mM (N5). B = no DNA. M = molecular weight marker.

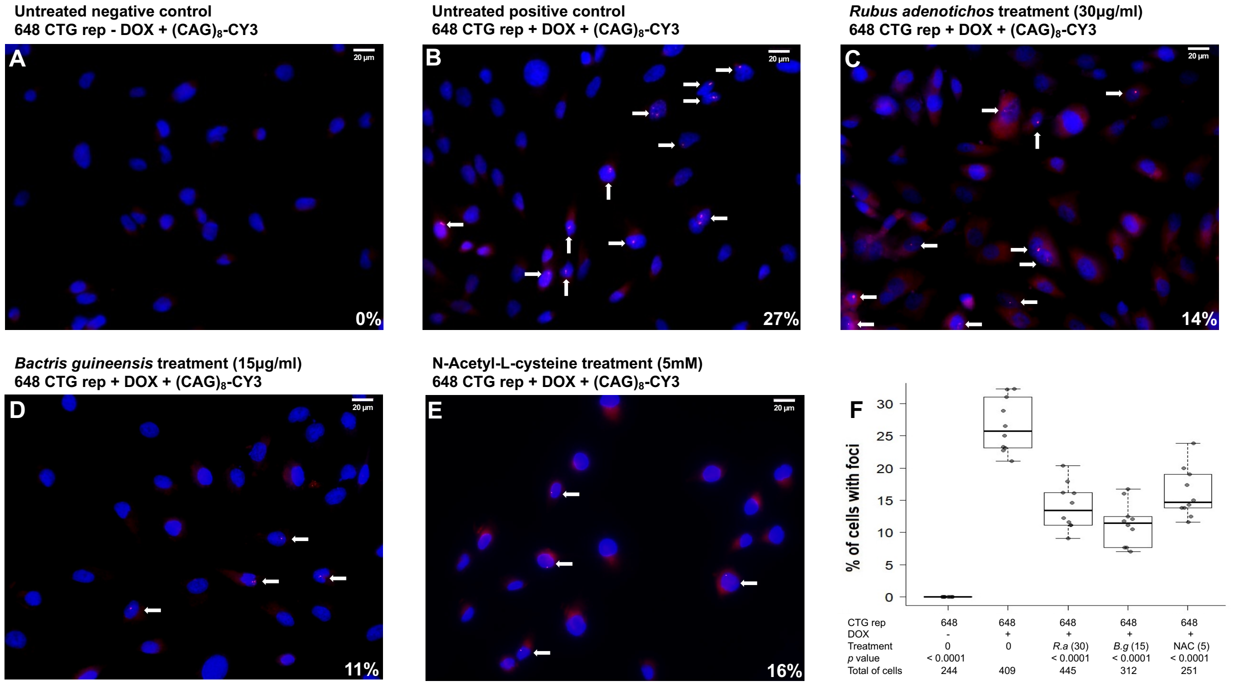

Figure S2. Foci formation and antioxidant treatment after induction. Cells were induced by 2 days, and after that, antioxidants were added to the culture and treated for 3 days. Foci detection and counting was performed as described in the main text. **A:** MIO-M1 $CTG_{(648)}$ uninduced cells (negative control). **B:** MIO-M1 $CTG_{(648)}$ induced cells (positive control) **C:** MIO-M1 $CTG_{(648)}$ induced cells treated with the highest concentration (30µg/ml) of polyphenol extracts from *Rubus adenotrichos*. **D:** MIO-M1 $CTG_{(648)}$ induced cells treated with the highest concentration (15µg/ml) of polyphenol extracts from *Bactris guineensis*. **E:** MIO-M1 $CTG_{(648)}$ induced cells treated with the highest concentration (5mM) of NAC. The percentage of cells with foci is indicated on each micrography (bottom right), with arrows pointing out the foci. **F:** Box plot showing the statistical analysis (ANOVA-Tukey), which indicates that the antioxidants significantly reduce the percentage of cells with foci in cultures where antioxidants were added after DOX induction, $p < 0.001$. *R.a* (30) = *Rubus adenotrichos* at 30µg/ml; *B.g* (15) = *Bactris guineensis* at 15µg/ml; NAC (5) = NAC at 5mM

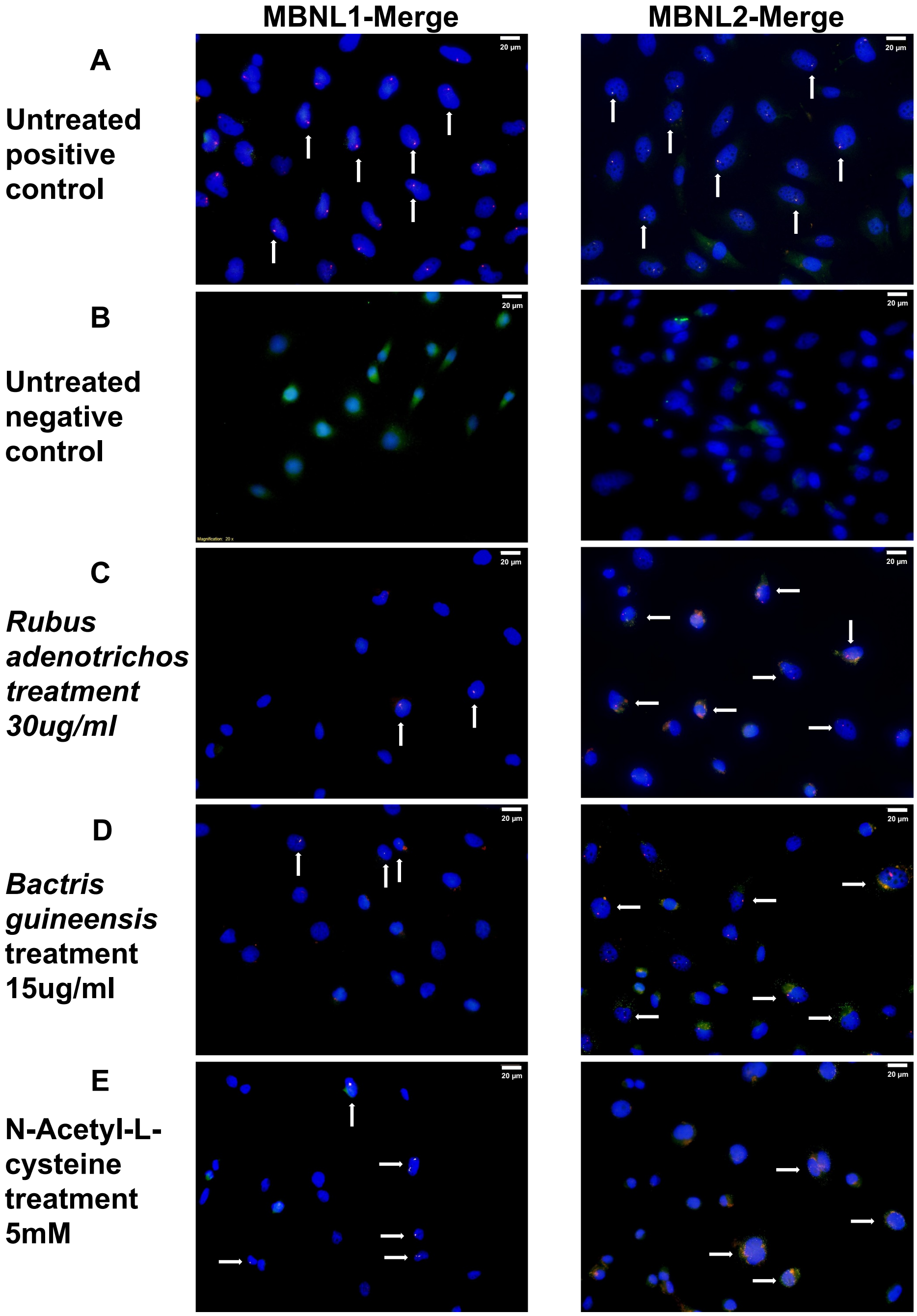

Figure S3. MBNL1/2 colocalization and antioxidant treatment. The panels show MBNL1 (left panels) and MBNL2 (right panels) foci colocalization in different culture conditions. A - MIO-M1 $CTG_{(648)}$ induced cells without antioxidant treatment. Colocalization is observed for both, MBNL1 and MBNL2. B - MIO-M1 $CTG_{(648)}$ uninduced cells without antioxidant treatment. Colocalization is not observed for both, MBNL1 and MBNL2. C - MIO-M1 $CTG_{(648)}$ induced cells treated with the highest concentration of polyphenol extracts from *Rubus adenotrichos*. Clearer colocalization is observed for MBNL1 but not for MBNL2. D - MIO-M1 $CTG_{(648)}$ induced cells treated with the highest concentration of polyphenol extracts from *Bactris guineensis*. Clearer colocalization is observed for MBNL1 but not for MBNL2. E - MIO-M1 $CTG_{(648)}$ induced cells treated with the highest concentration of N-acetyl cysteine. Clearer colocalization is observed for MBNL1 but not for MBNL2. Arrows show some of the signals corresponding to colocalization or the absence of colocalization.

Foci-MBNL1 colocalization in MIO-M1 $CTG_{(648)}$ glial cells and antioxidant treatment

Untreated induced MIO-M1 $CTG_{(648)}$ glial cells – positive control

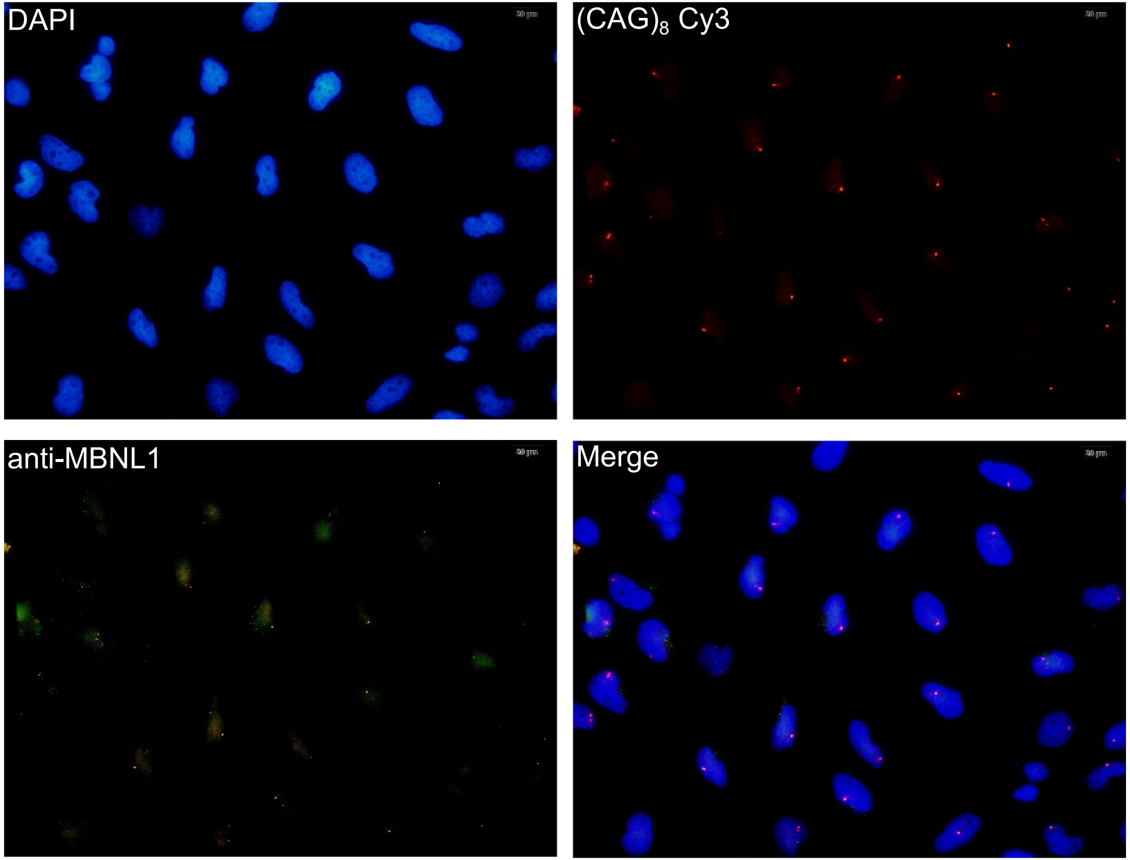

Untreated uninduced MIO-M1 CTG$_{(648)}$ glial cells – negative control

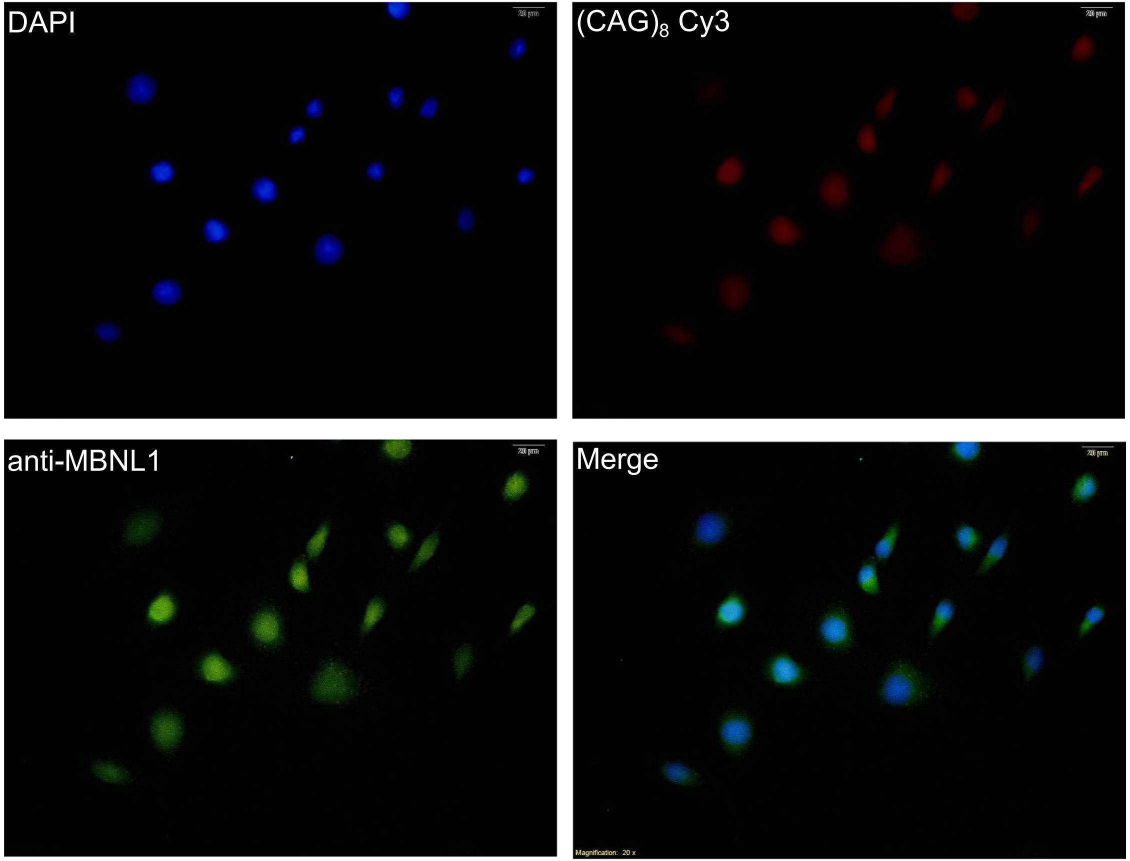

Treated induced MIO-M1 CTG$_{(648)}$ glial cells – polyphenol extracts from *Rubus adenotrichos* at 7.5 µg/mL

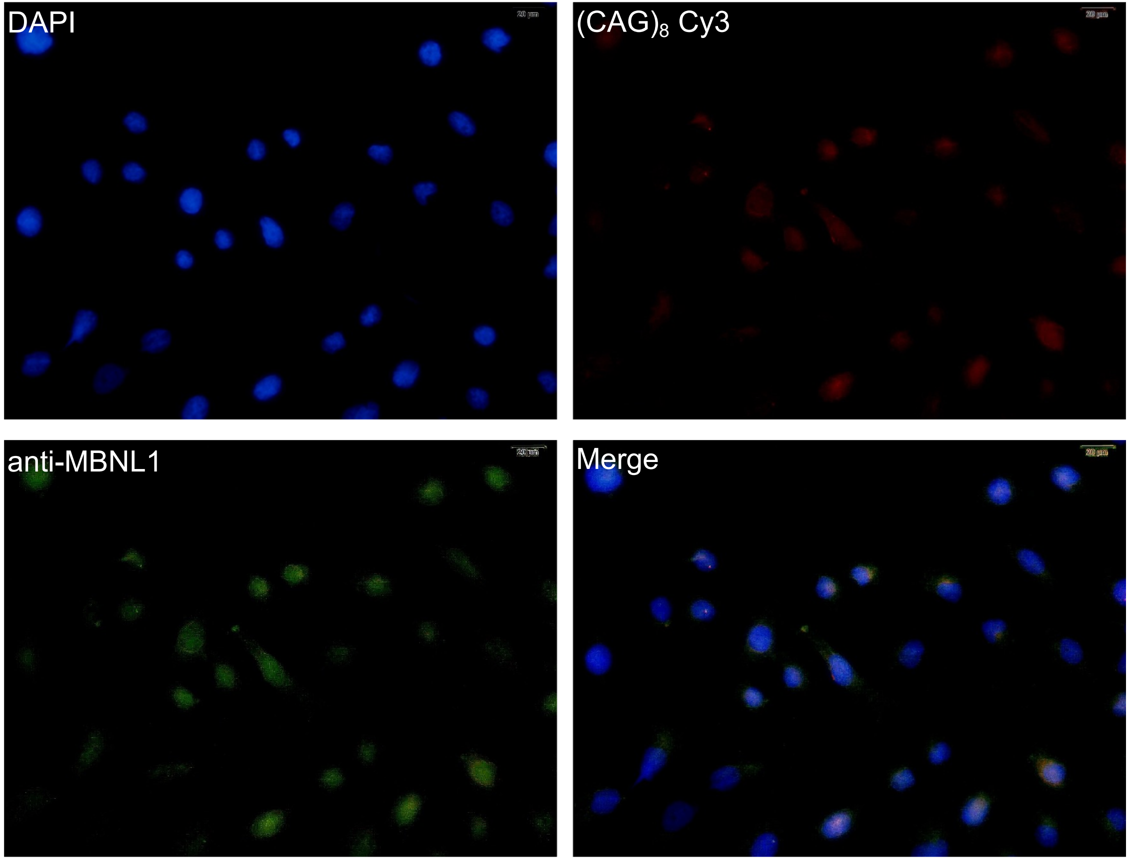

Treated induced MIO-M1 $CTG_{(648)}$ glial cells – polyphenol extracts from *Rubus adenotrichos* at 15 µg/mL

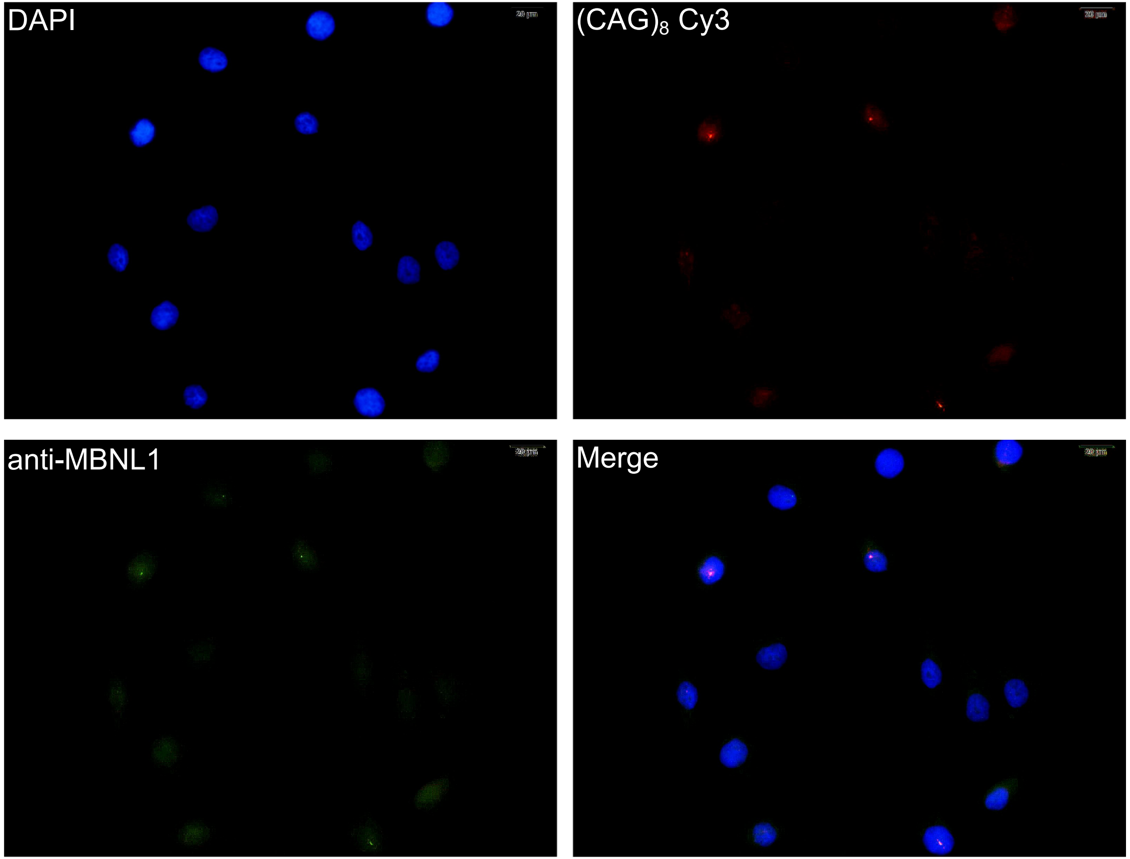

Treated induced MIO-M1 $CTG_{(648)}$ glial cells – polyphenol extracts from *Rubus adenotrichos* at 30 µg/mL

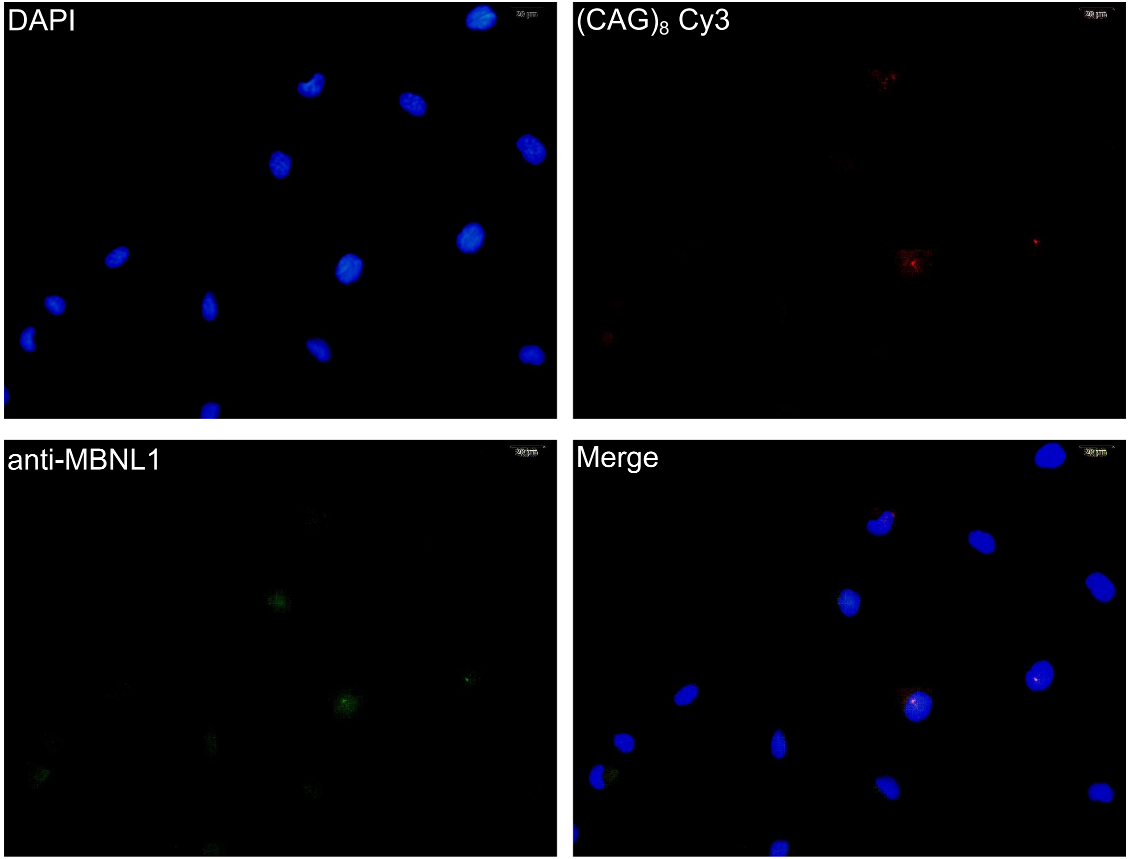

Treated induced MIO-M1 $CTG_{(648)}$ glial cells – polyphenol extracts from *Bactris guineensis* at 5 µg/mL

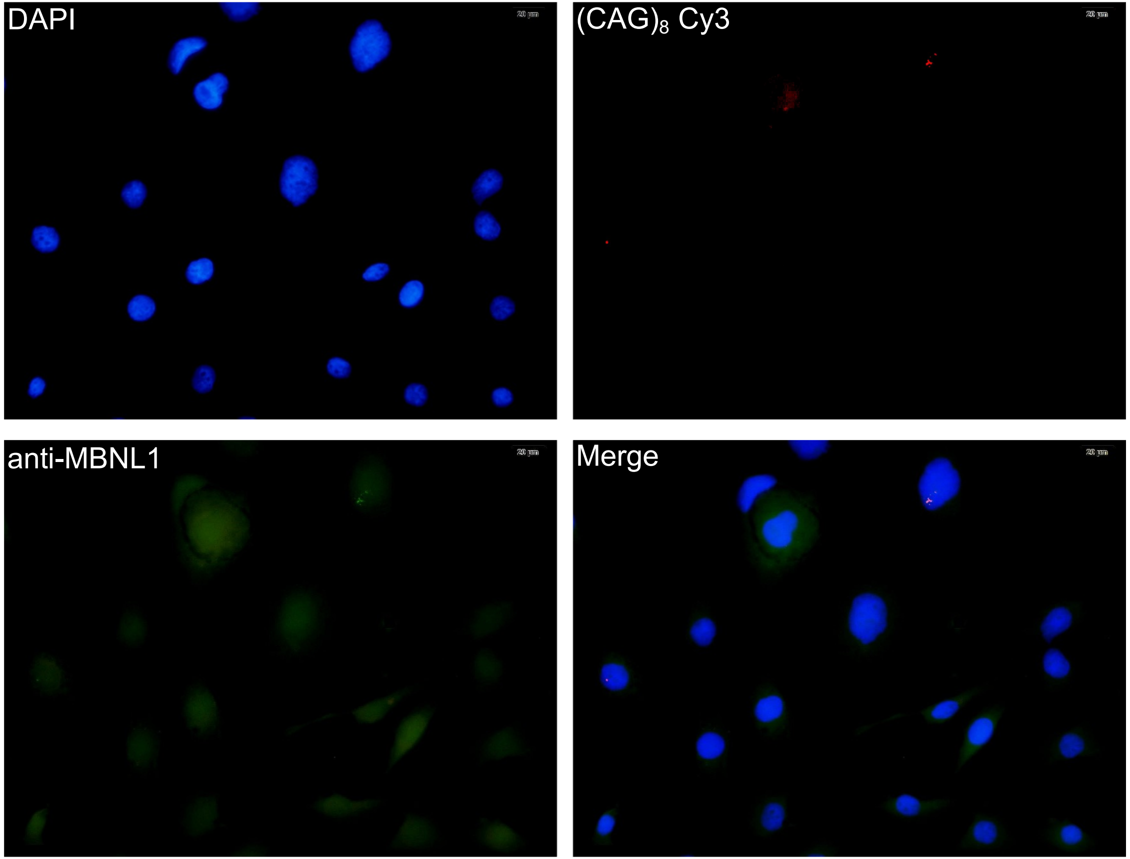

Treated induced MIO-M1 $CTG_{(648)}$ glial cells – polyphenol extracts from *Bactris guineensis* at 10 µg/mL

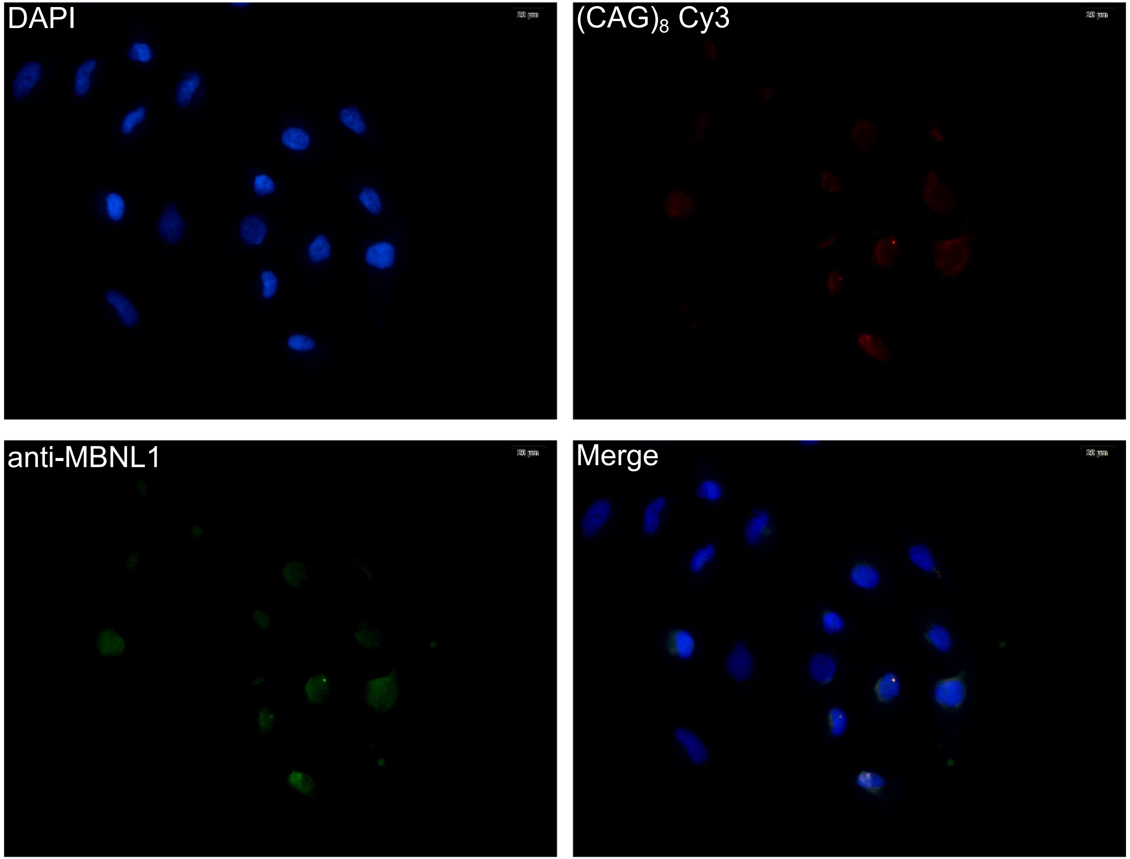

Treated induced MIO-M1 CTG$_{(648)}$ glial cells – polyphenol extracts from *Bactris guineensis* at 15 µg/mL

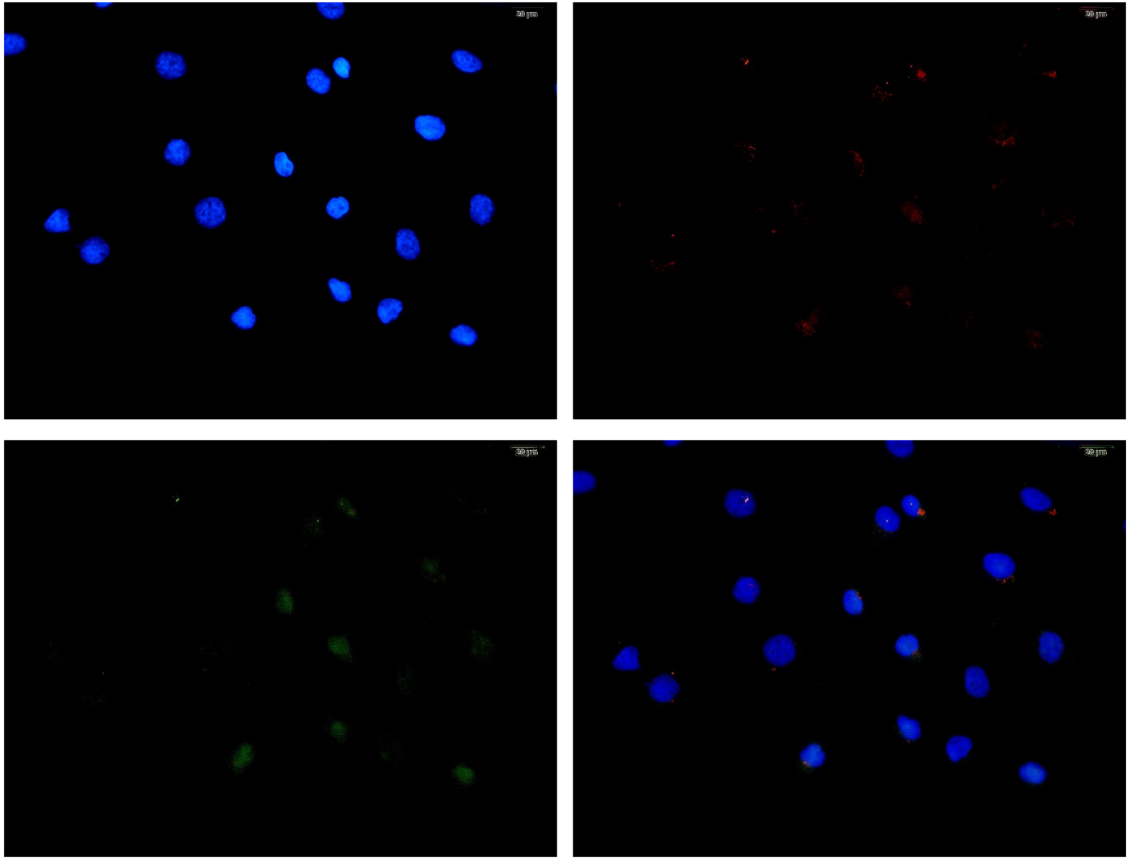

Treated induced MIO-M1 CTG$_{(648)}$ glial cells – N-acetyl cysteine 1 mM

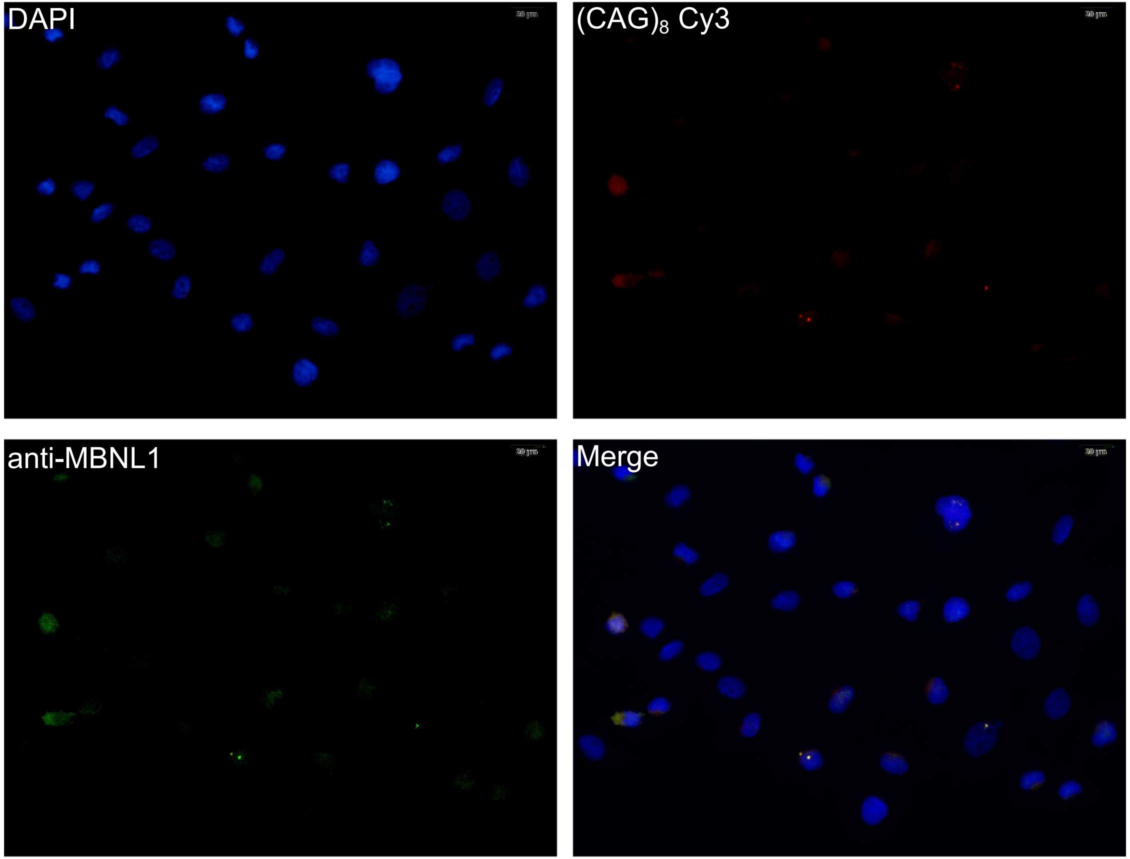

Treated induced MIO-M1 CTG$_{(648)}$ glial cells – N-acetyl cysteine 3 mM

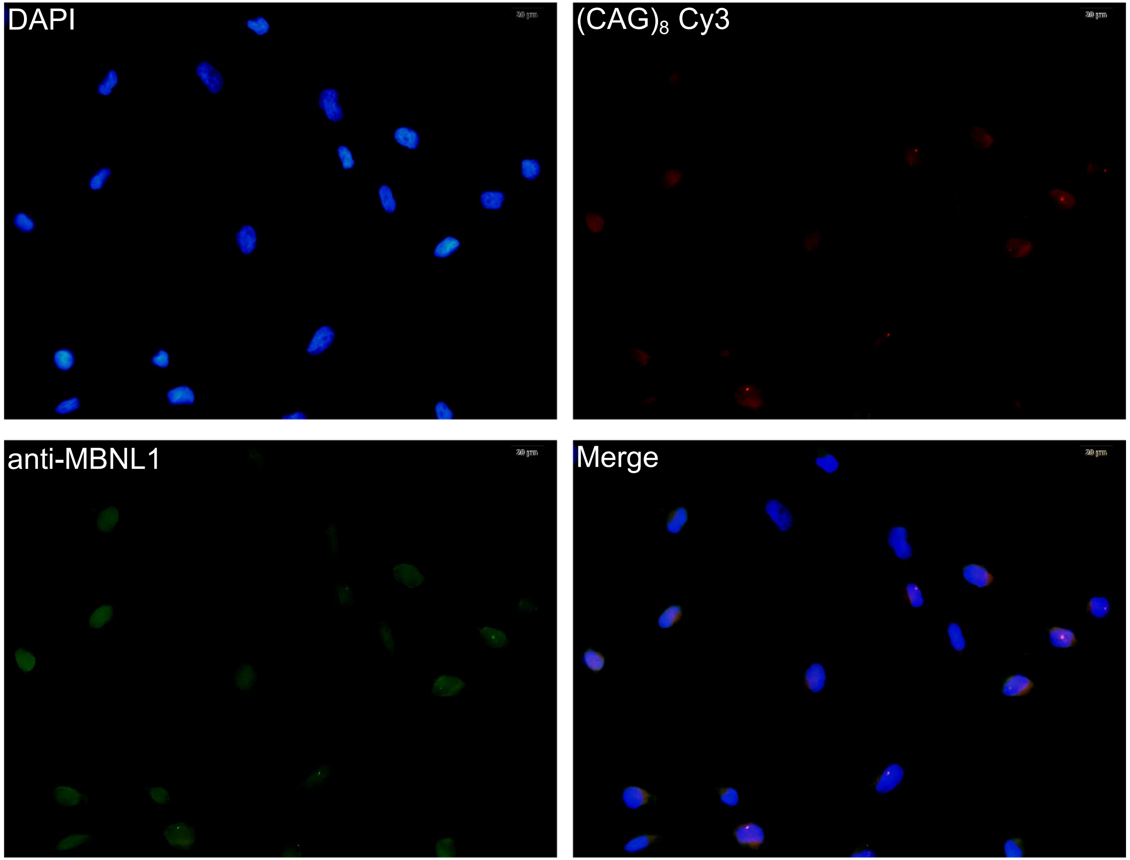

Treated induced MIO-M1 CTG$_{(648)}$ glial cells – N-acetyl cysteine 5 mM

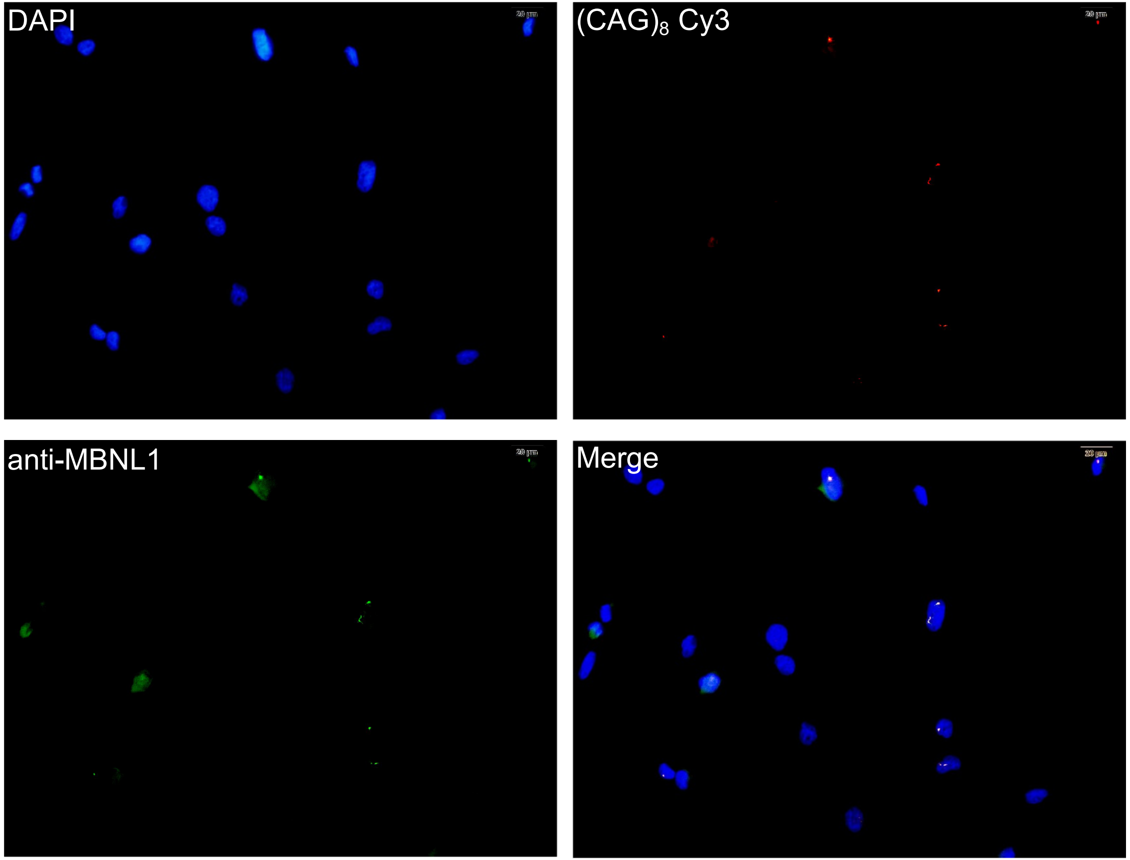

Foci-MBNL2 colocalization in MIO-M1 $CTG_{(648)}$ glial cells and antioxidant treatment

Untreated induced MIO-M1 $CTG_{(648)}$ glial cells – positive control

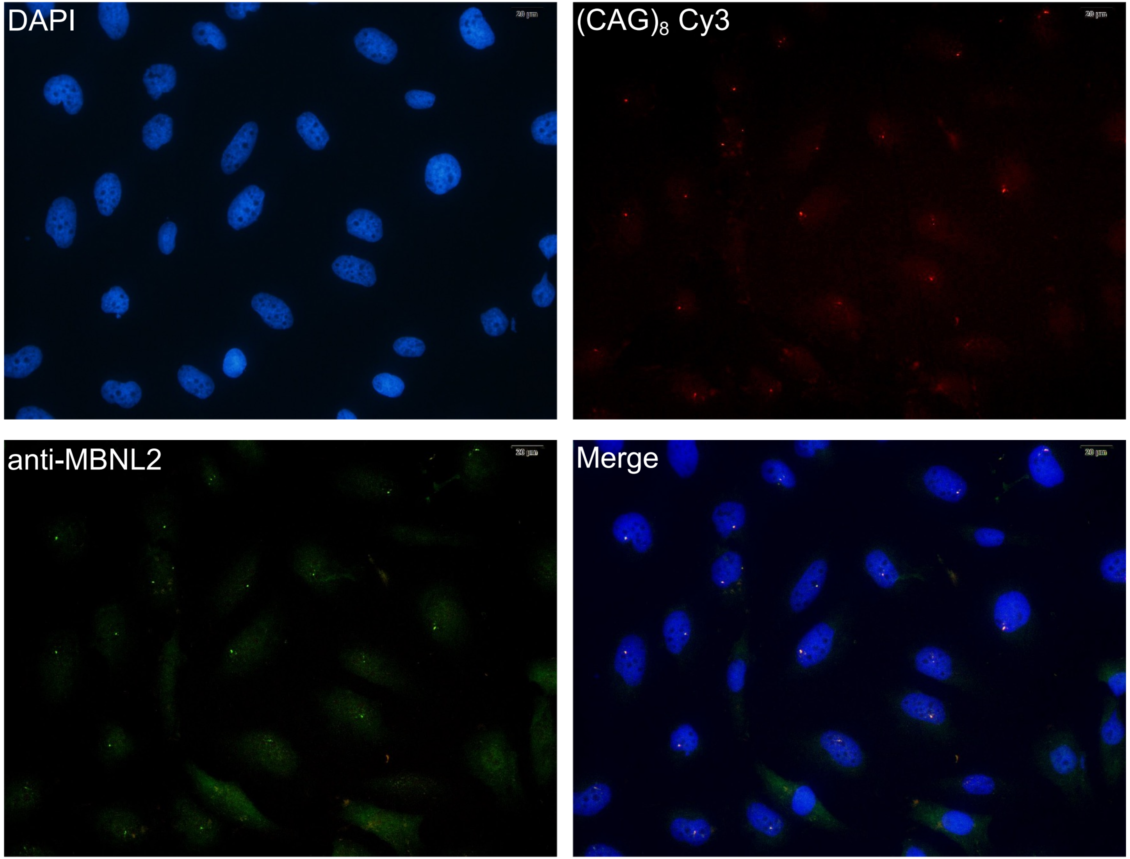

Untreated uninduced MIO-M1 $CTG_{(648)}$ glial cells – negative control

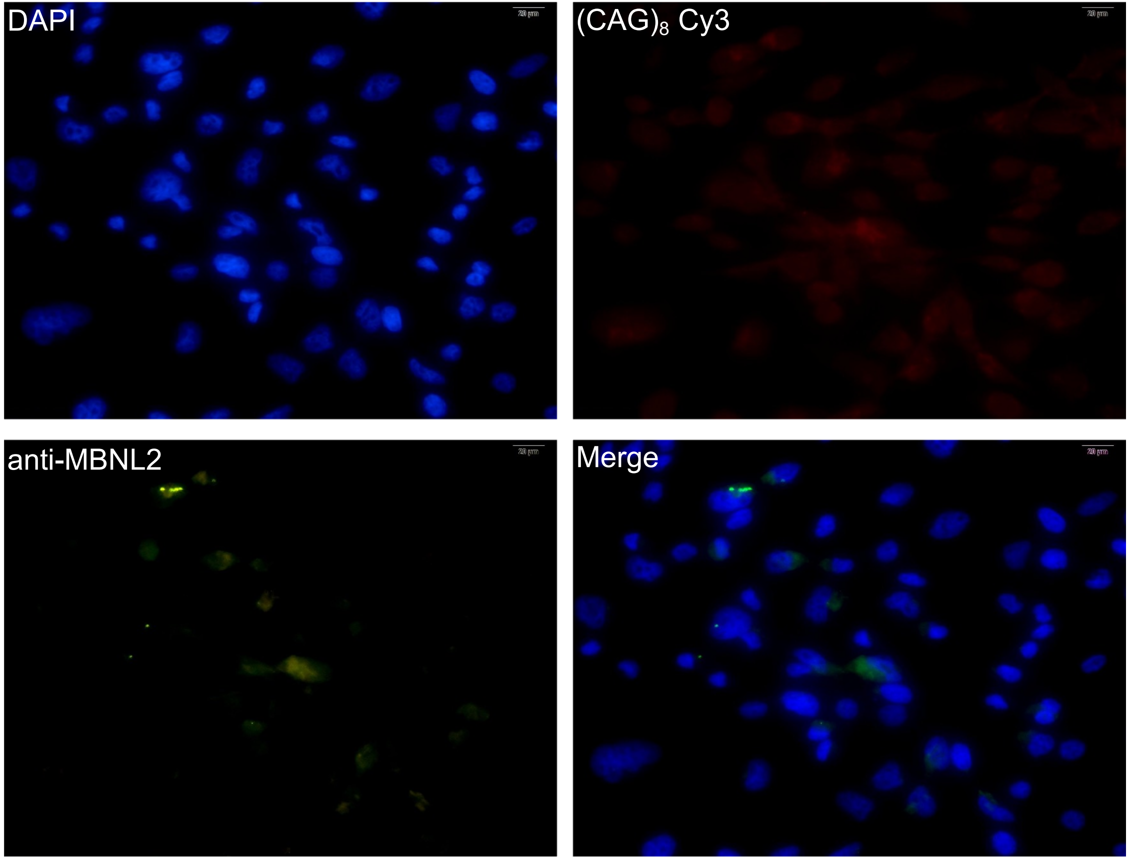

Treated induced MIO-M1 $CTG_{(648)}$ glial cells – polyphenol extracts from *Rubus adenotrichos* at 7.5 µg/mL

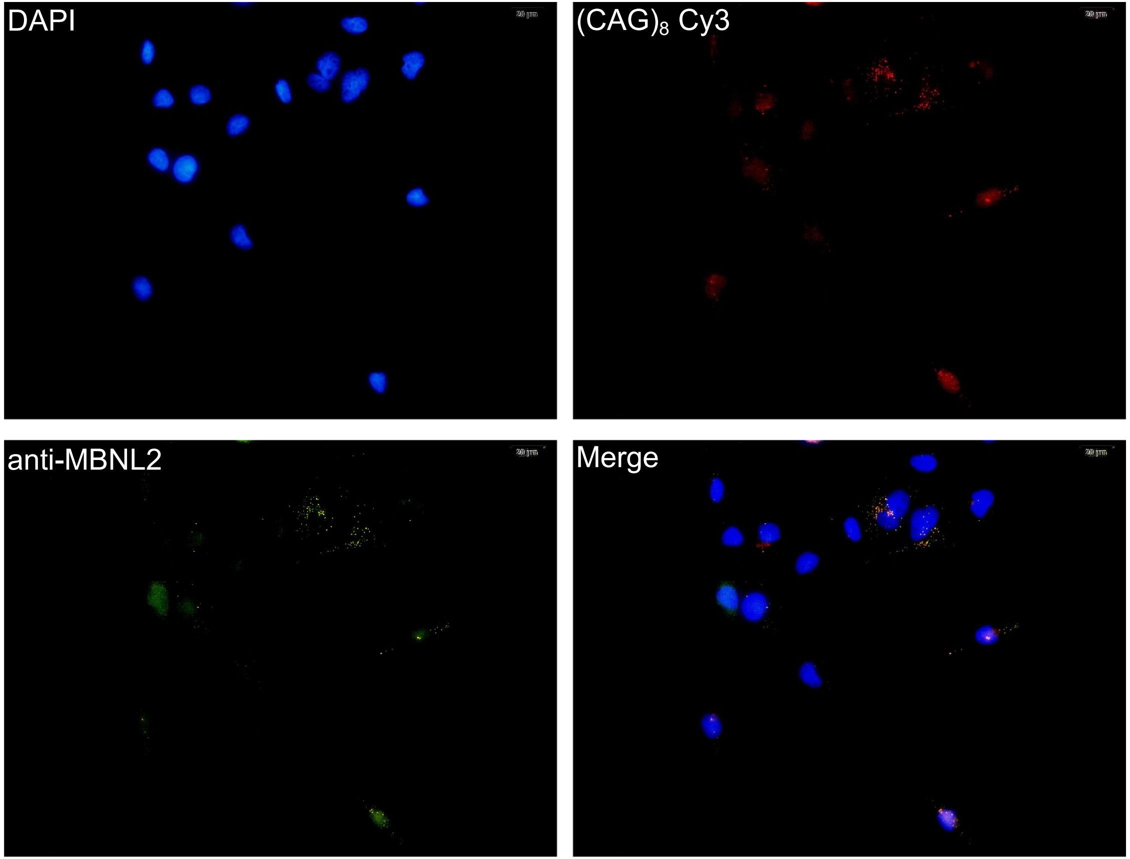

Treated induced MIO-M1 $CTG_{(648)}$ glial cells – polyphenol extracts from *Rubus adenotrichos* at 15 µg/mL

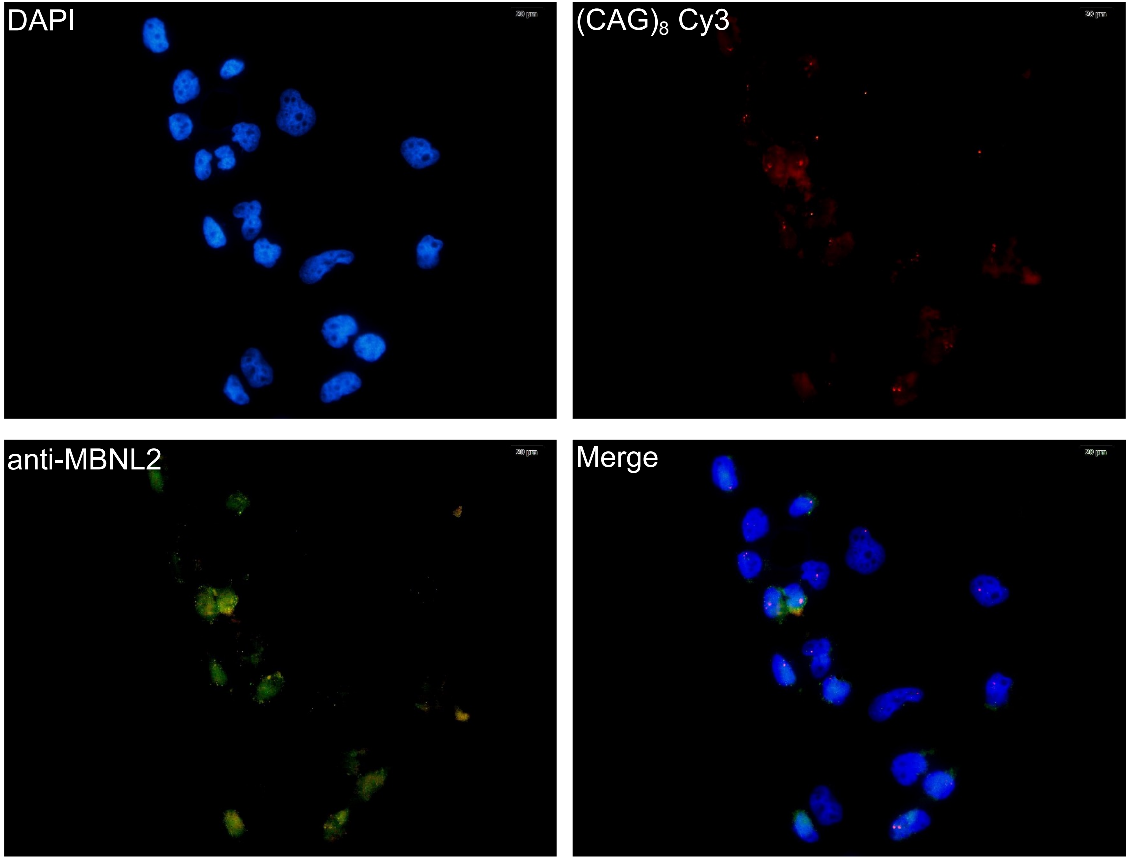

Treated induced MIO-M1 CTG$_{(648)}$ glial cells – polyphenol extracts from *Rubus adenotrichos* at 30 µg/mL

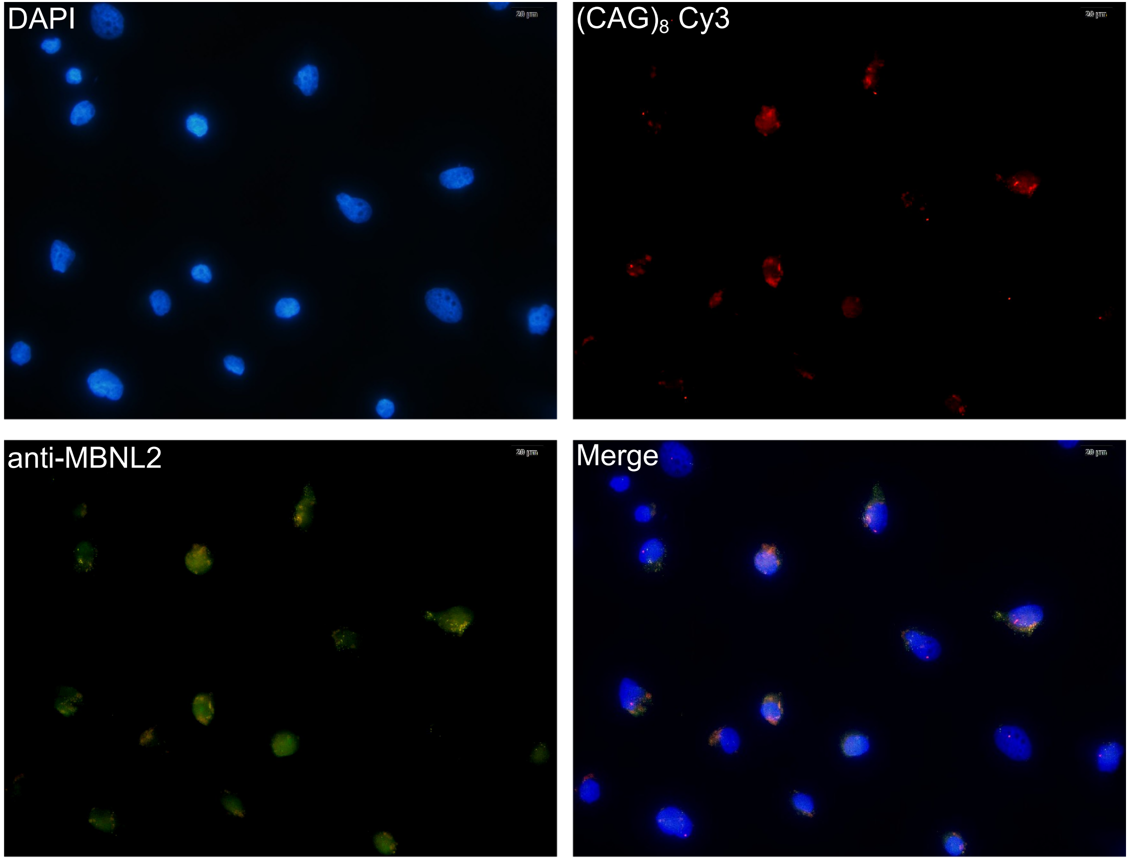

Treated induced MIO-M1 CTG$_{(648)}$ glial cells – polyphenol extracts from *Bactris guineensis* at 5 µg/mL

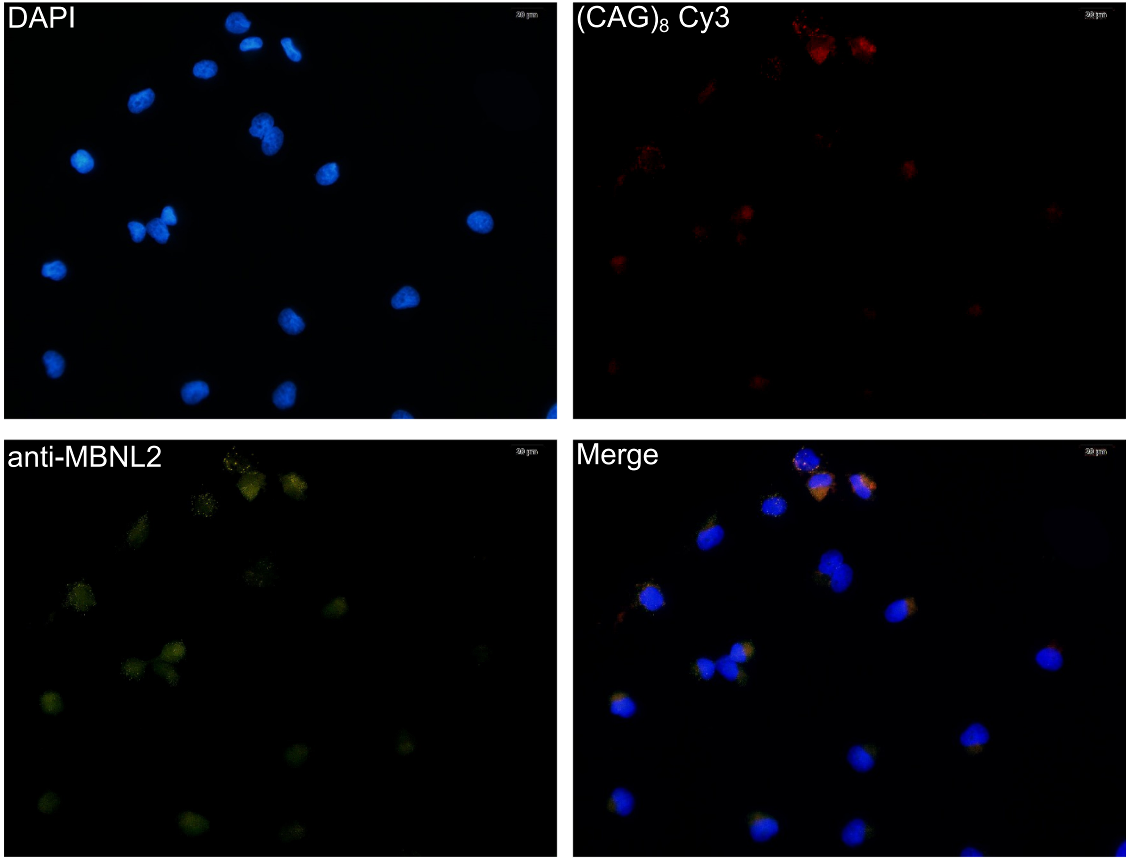

Treated induced MIO-M1 $CTG_{(648)}$ glial cells – polyphenol extracts from *Bactris guineensis* at 10 µg/mL

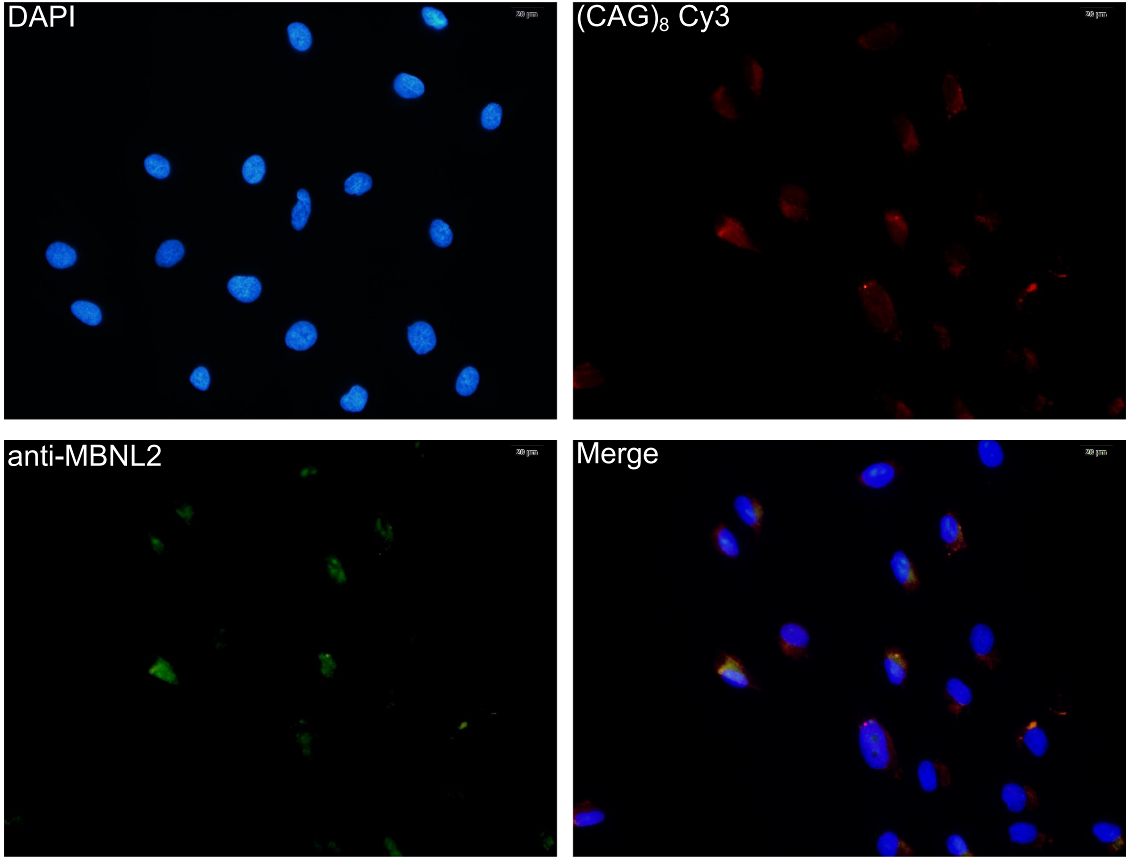

Treated induced MIO-M1 CTG$_{(648)}$ glial cells – polyphenol extracts from *Bactris guineensis* at 15 µg/mL

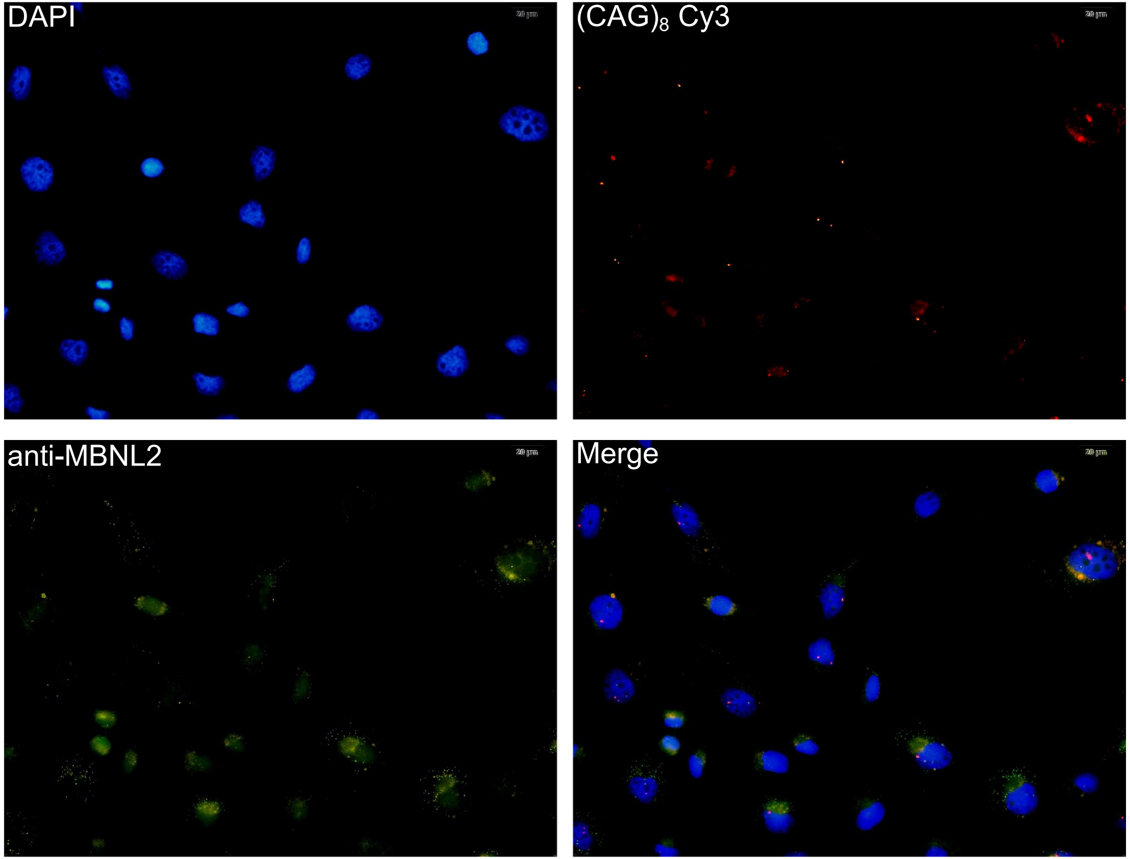

Treated induced MIO-M1 $CTG_{(648)}$ glial cells – N-acetyl cysteine 1 mM

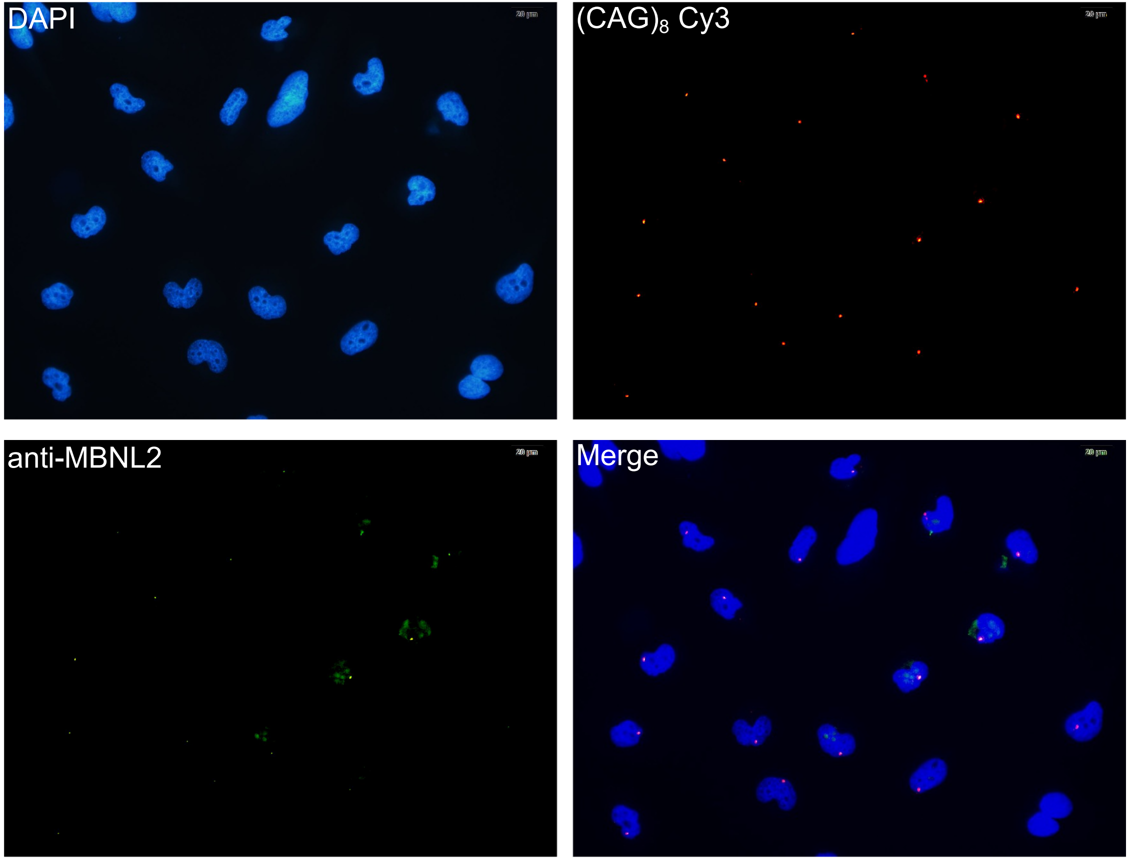

Treated induced MIO-M1 CTG$_{(648)}$ glial cells – N-acetyl cysteine 3 mM

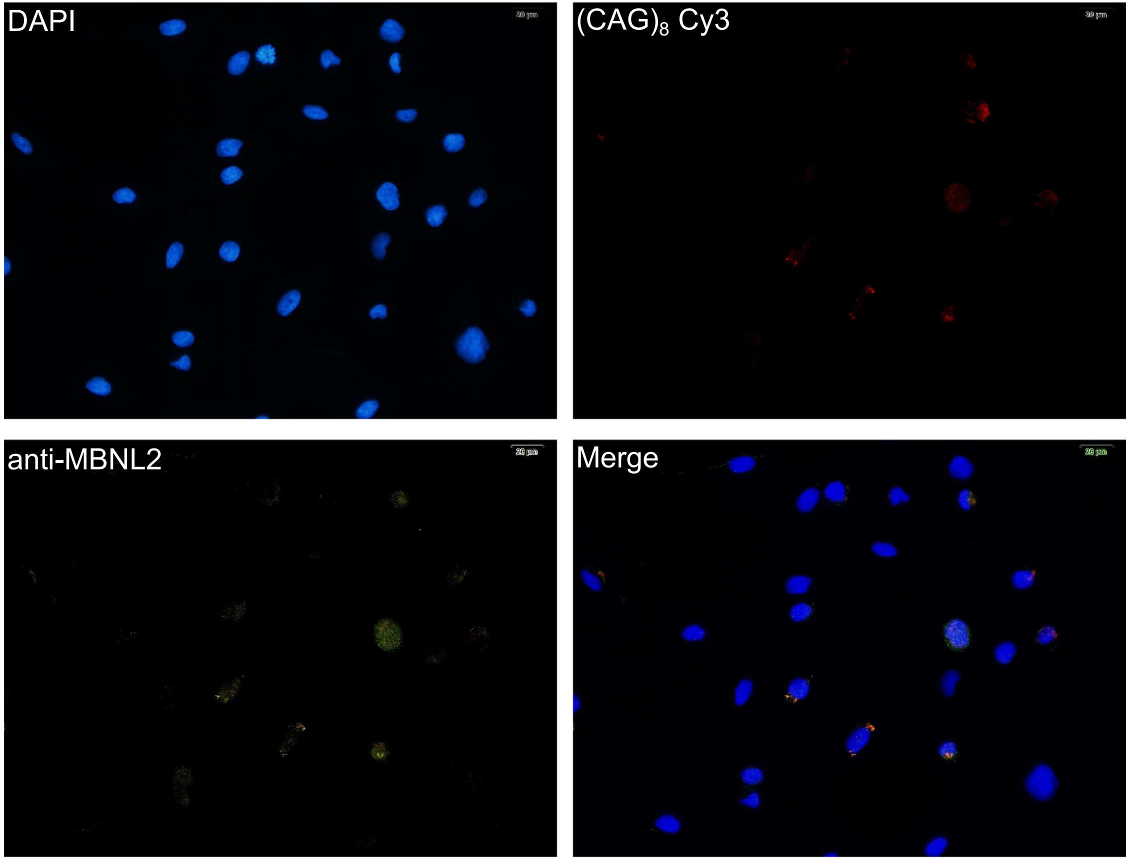

Treated induced MIO-M1 $CTG_{(648)}$ glial cells – N-acetyl cysteine 5 mM

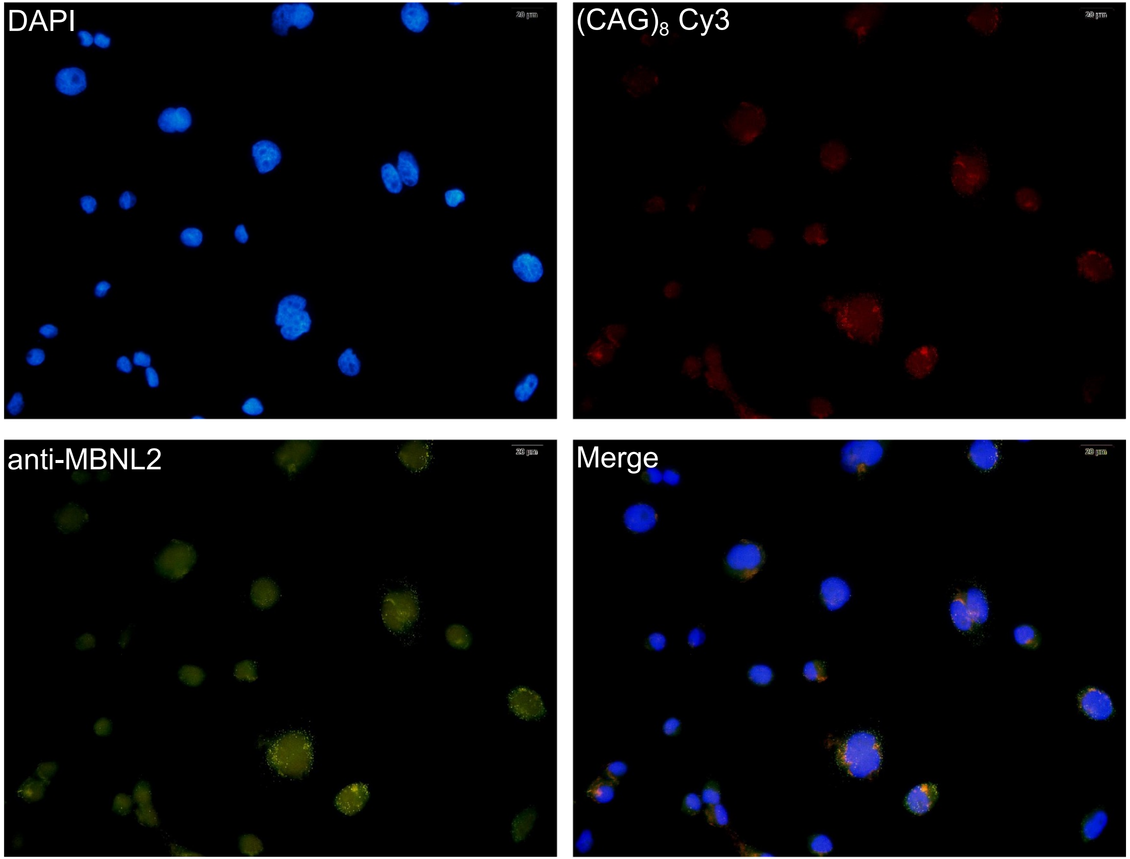

Ethidium bromide agarose geles

POLR2A

1st PCR *2nd PCR*

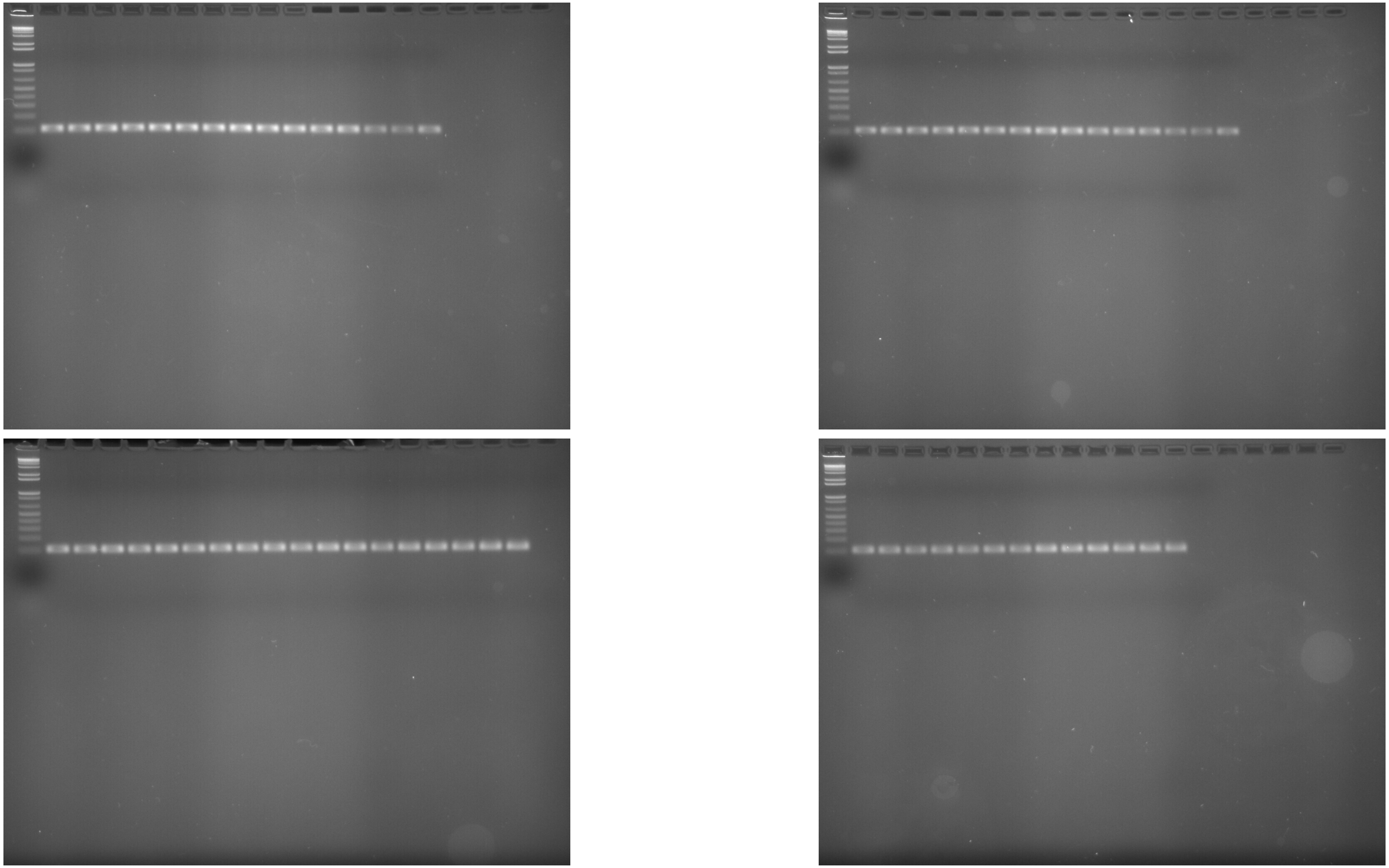

MBNL1 Exon 7

Bactris guineensis

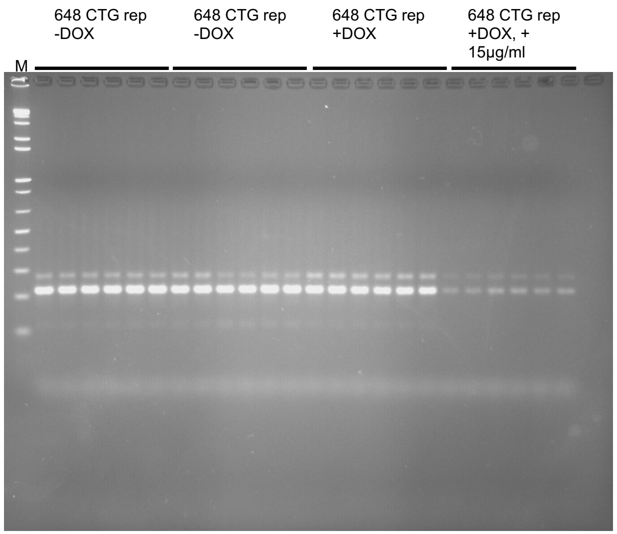

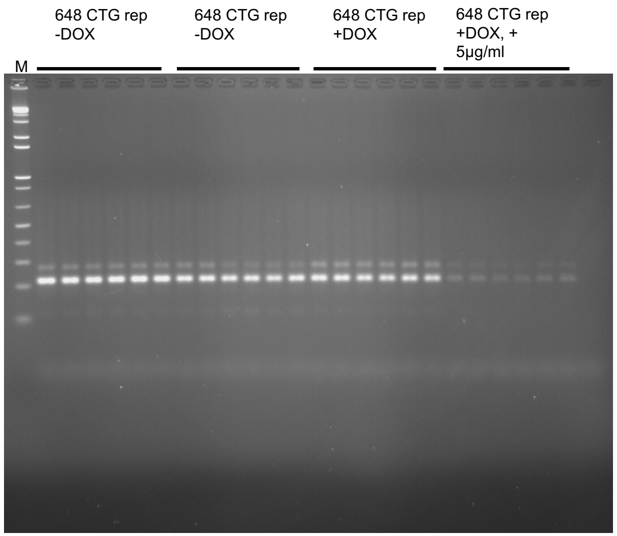

Rubus adenotichos

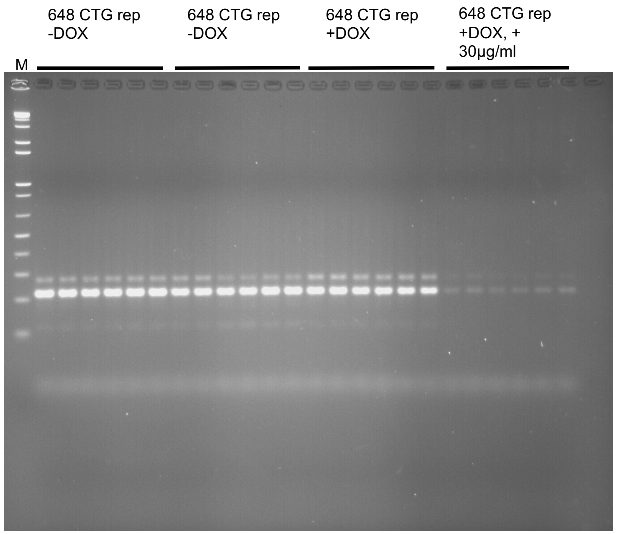

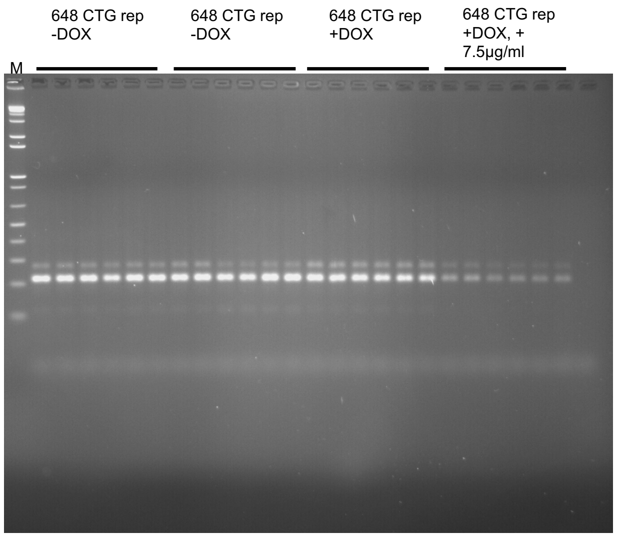

N-acetyl cysteine

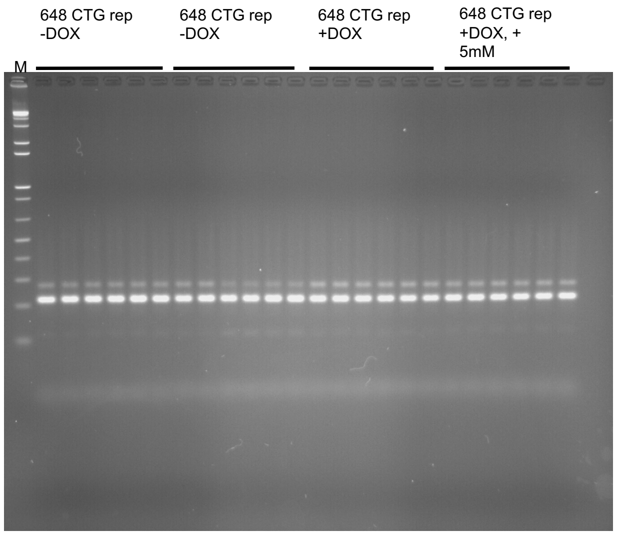

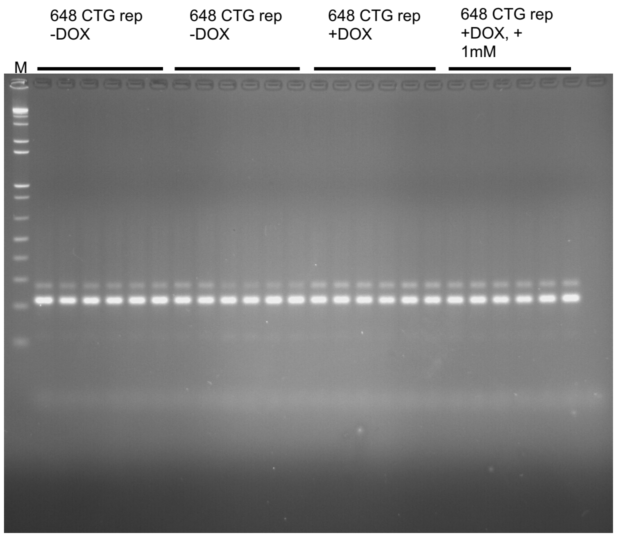

MBNL2 Exon 7

Bactris guineensis

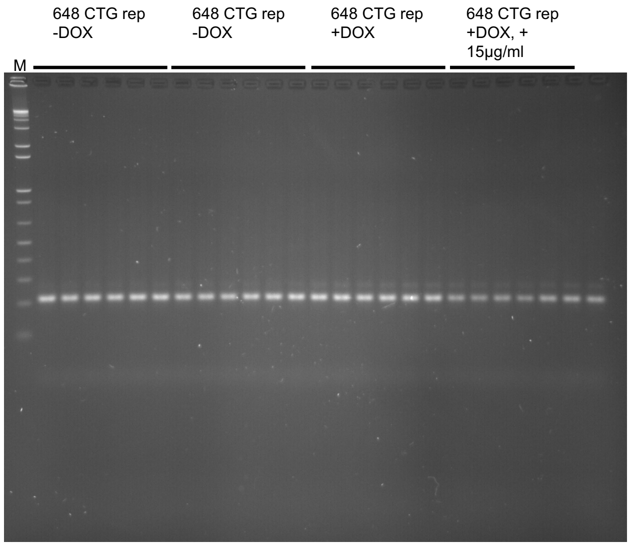

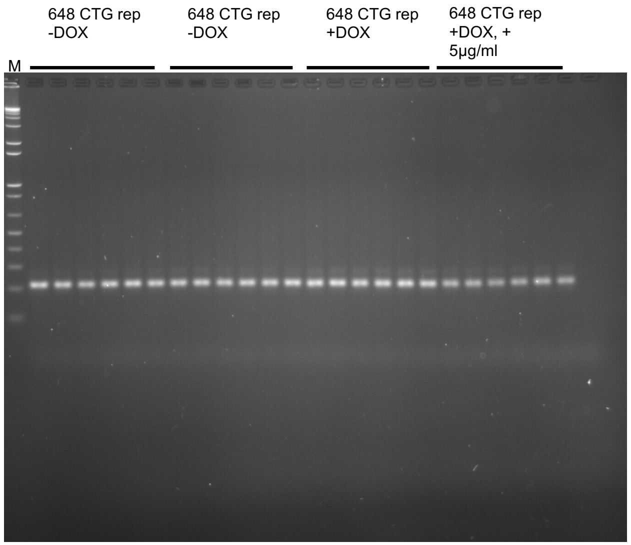

Rubus adenotichos

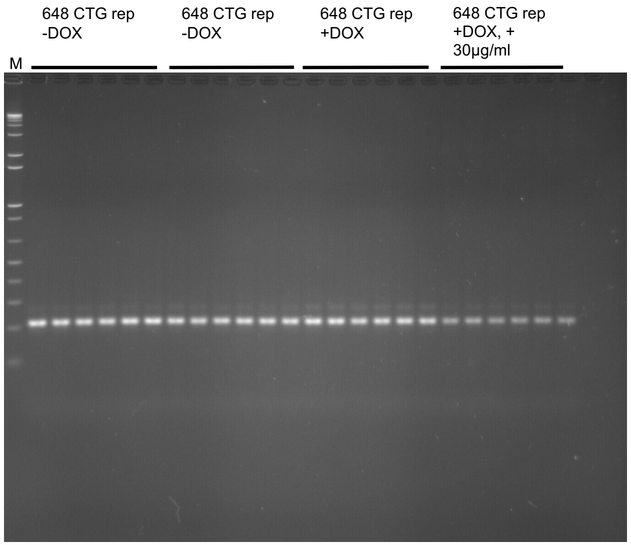

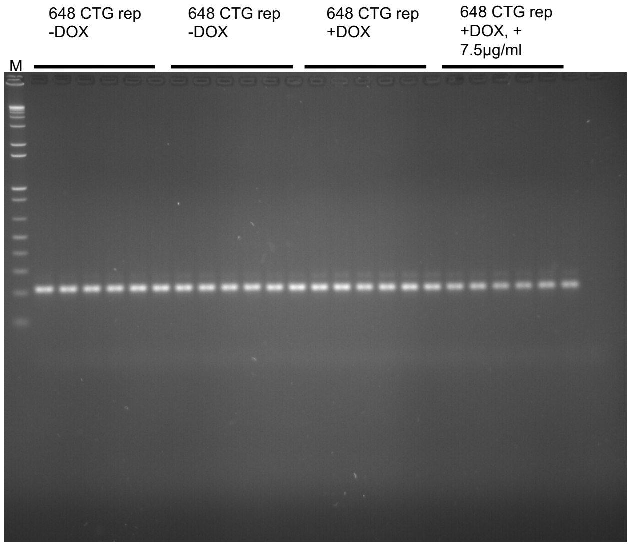

N-acetyl cysteine

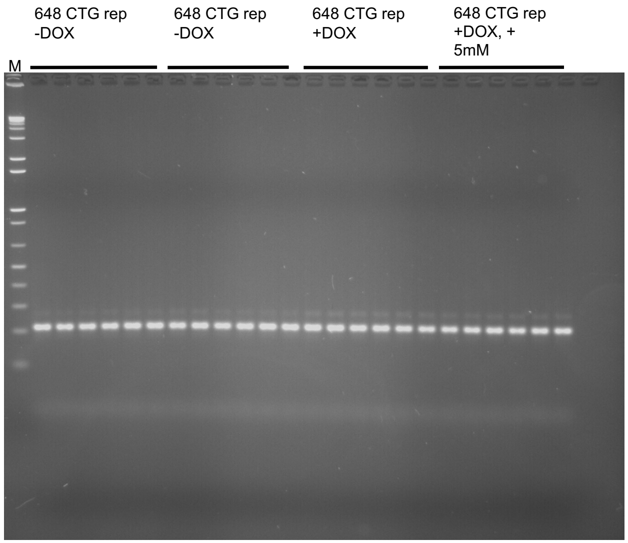

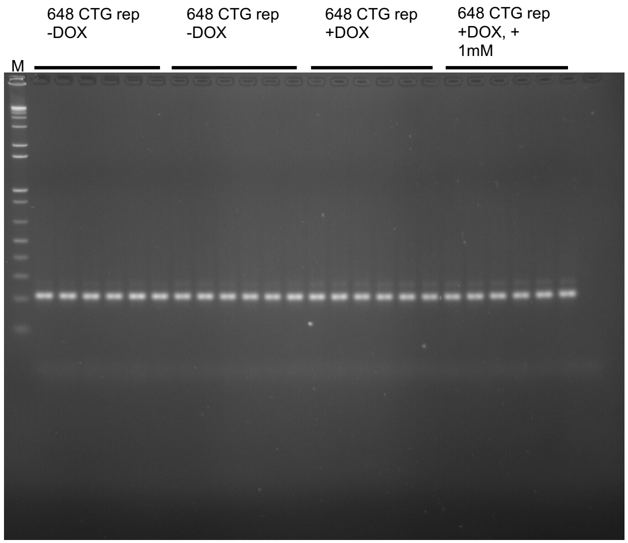

APP Exon 8

Bactris guineensis

648 CTG rep -DOX | 648 CTG rep -DOX | 648 CTG rep +DOX | 648 CTG rep +DOX, + 15µg/ml

M

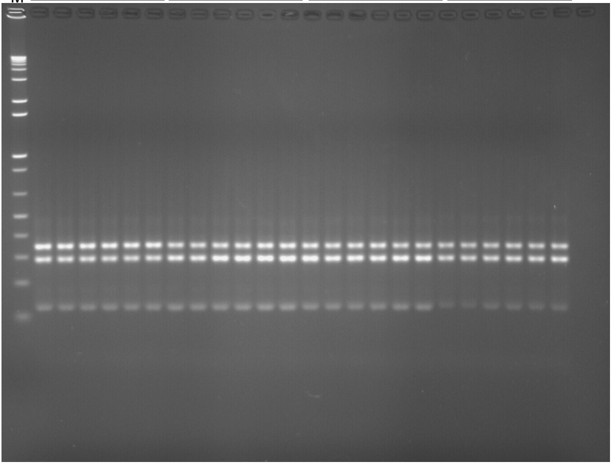

648 CTG rep -DOX | 648 CTG rep -DOX | 648 CTG rep +DOX | 648 CTG rep +DOX, + 5µg/ml

M

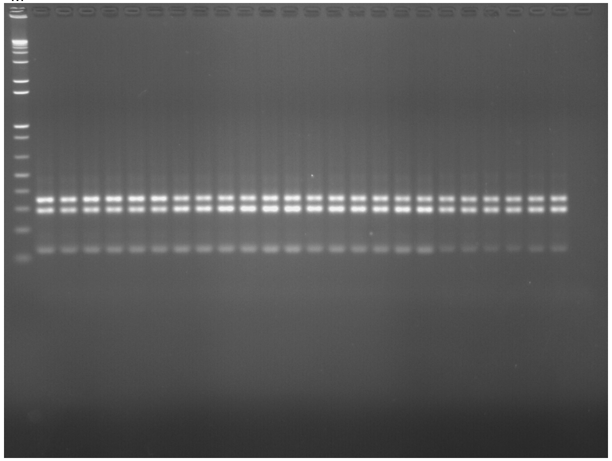

Rubus adenotichos

648 CTG rep -DOX | 648 CTG rep -DOX | 648 CTG rep +DOX | 648 CTG rep +DOX, + 30µg/ml

M

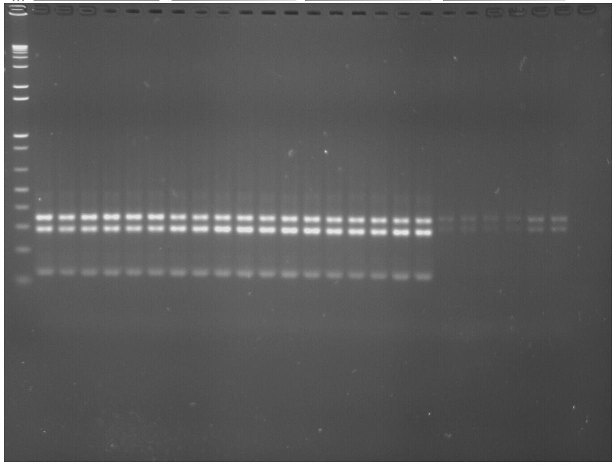

648 CTG rep -DOX | 648 CTG rep -DOX | 648 CTG rep +DOX | 648 CTG rep +DOX, + 7.5µg/ml

M

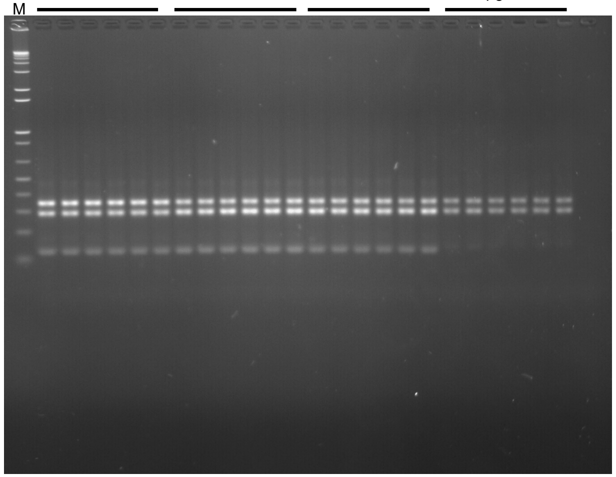

N-acetyl cysteine

648 CTG rep -DOX | 648 CTG rep -DOX | 648 CTG rep +DOX | 648 CTG rep +DOX, + 5mM

M

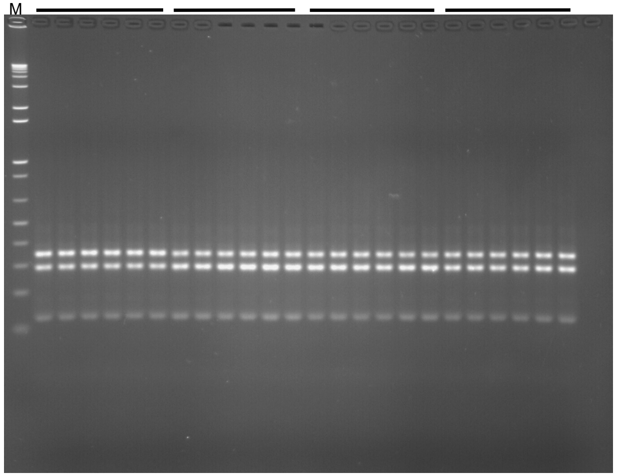

648 CTG rep -DOX | 648 CTG rep -DOX | 648 CTG rep +DOX | 648 CTG rep +DOX, + 1mM

M

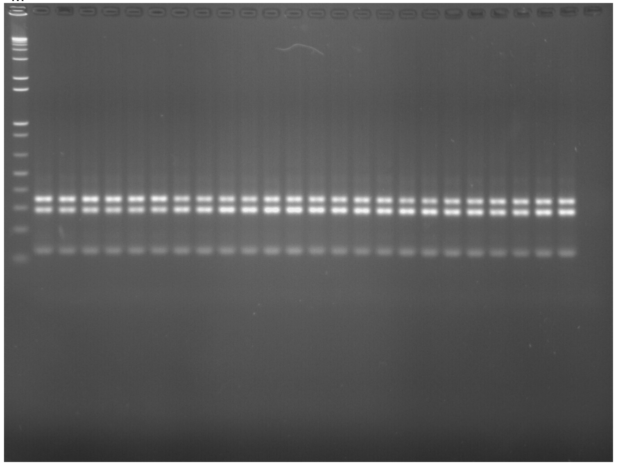

ITGA6 Exon 27

Bactris guineensis

648 CTG rep -DOX | 648 CTG rep -DOX | 648 CTG rep +DOX | 648 CTG rep +DOX, + 15µg/ml

M

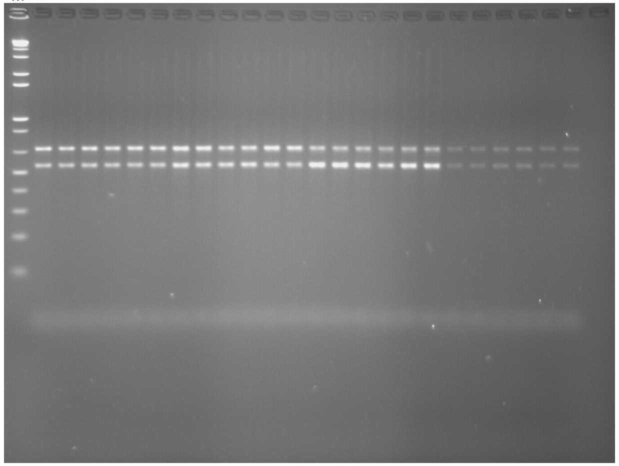

648 CTG rep -DOX | 648 CTG rep -DOX | 648 CTG rep +DOX | 648 CTG rep +DOX, + 5µg/ml

M

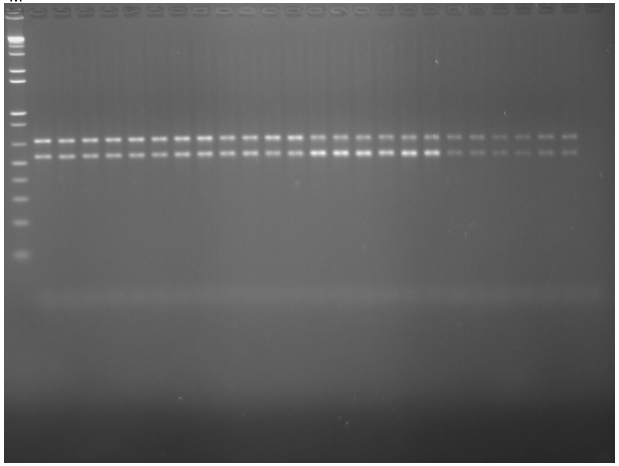

Rubus adenotichos

648 CTG rep -DOX | 648 CTG rep -DOX | 648 CTG rep +DOX | 648 CTG rep +DOX, + 30µg/ml

M

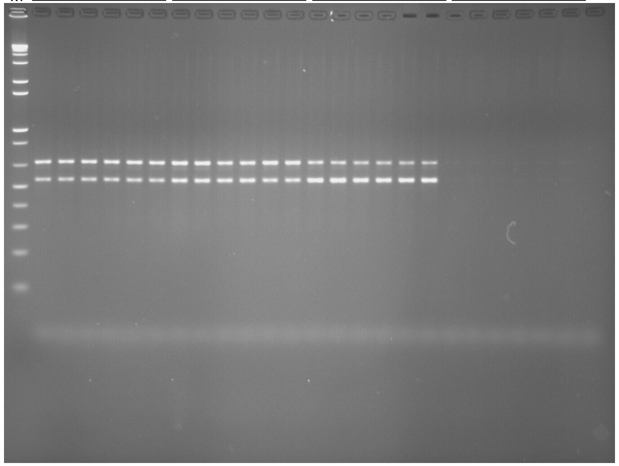

648 CTG rep -DOX | 648 CTG rep -DOX | 648 CTG rep +DOX | 648 CTG rep +DOX, + 7.5µg/ml

M

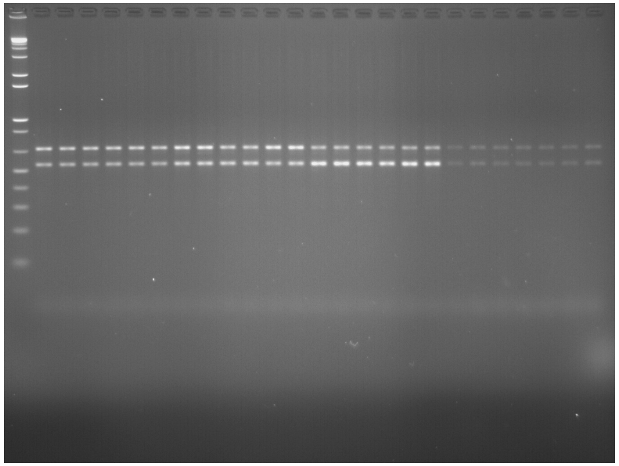

N-acetyl cysteine

648 CTG rep -DOX | 648 CTG rep -DOX | 648 CTG rep +DOX | 648 CTG rep +DOX, + 5mM

M

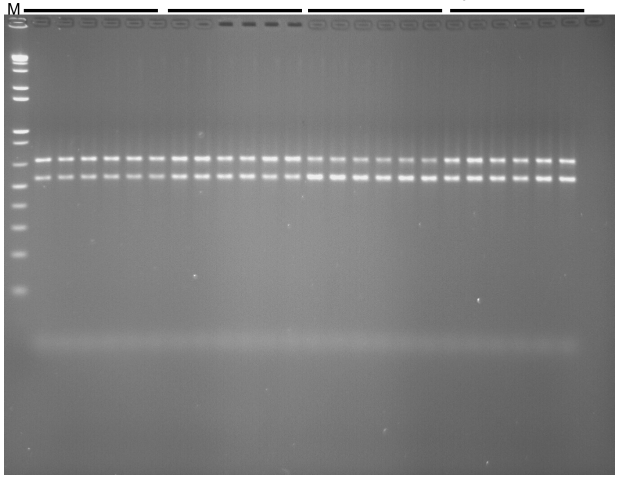

648 CTG rep -DOX | 648 CTG rep -DOX | 648 CTG rep +DOX | 648 CTG rep +DOX, + 1mM

M

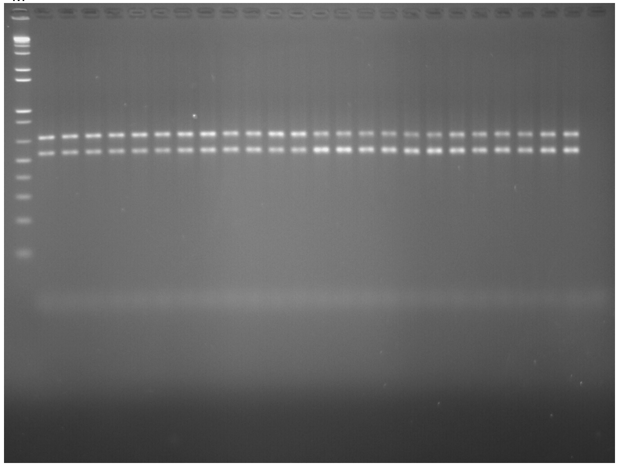

SORBS1 Exon 30

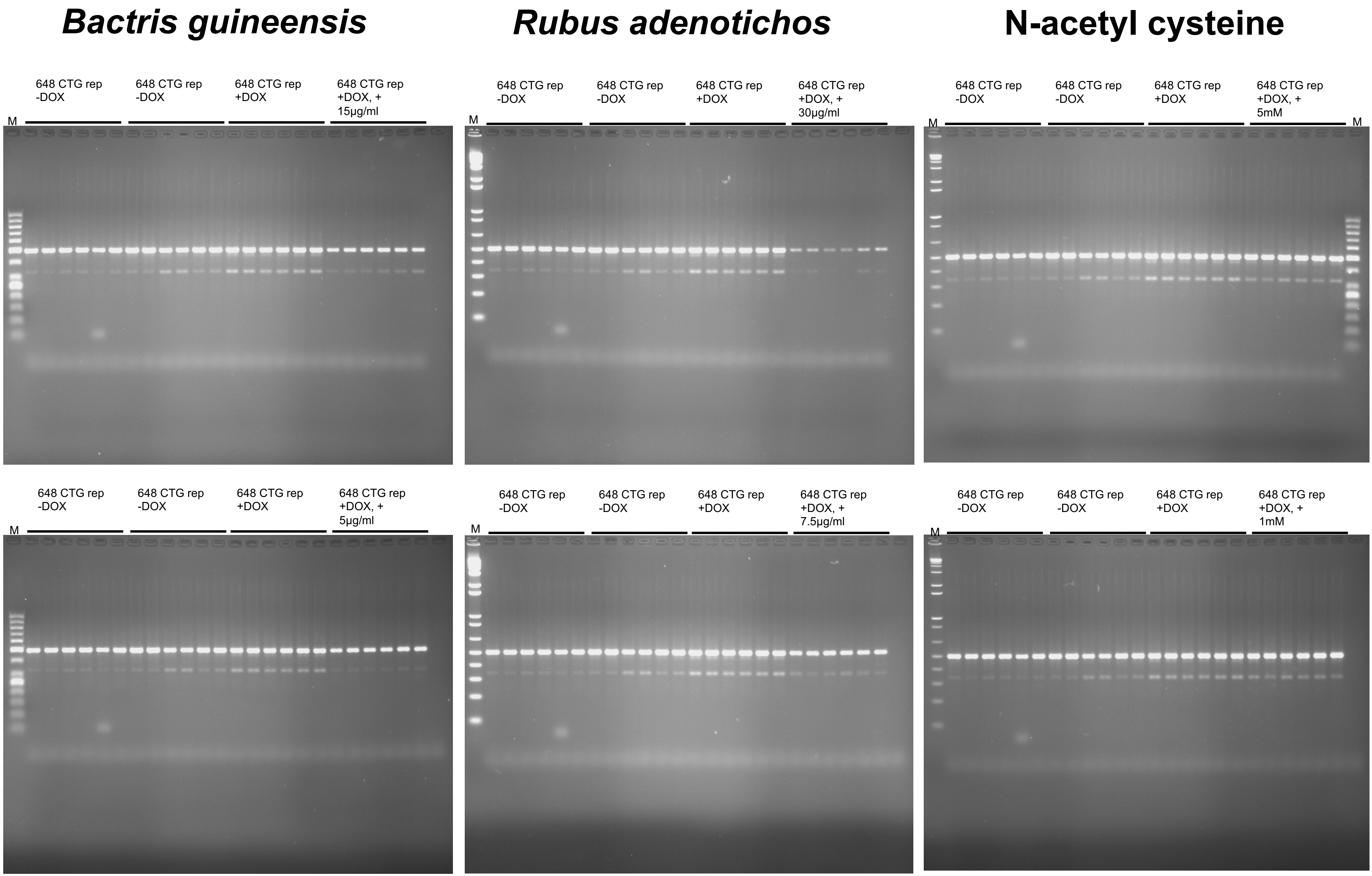

Supplement: Supplementary file 1 [file antioxidants-14-00260-s001.zip › antioxidants-3390468-supplementary.pdf]
